# Lipid Nanoparticle (LNP) Delivery Carrier-Assisted Targeted Controlled Release mRNA Vaccines in Tumor Immunity

**DOI:** 10.3390/vaccines12020186

**Published:** 2024-02-12

**Authors:** Liusheng Wu, Xiaoqiang Li, Xinye Qian, Shuang Wang, Jixian Liu, Jun Yan

**Affiliations:** 1 Center of Hepatobiliary Pancreatic Disease, Beijing Tsinghua Changgung Hospital, School of Medicine, Tsinghua University, Beijing 100084, China; wuliusheng852@126.com (L.W.); hotchqian@126.com (X.Q.); w-hu21@mails.tsinghua.edu.cn (S.W.); 2 Yong Loo Lin School of Medicine, National University of Singapore, Singapore 119077, Singapore; 3 Department of Thoracic Surgery, Peking University Shenzhen Hospital, Shenzhen 518036, China; dr.lixiaoqiang@gmail.com

**Keywords:** lipid nanoparticles (LNPs), mRNA vaccine, tumor immunity, delivery carrier, review

## Abstract

In recent years, lipid nanoparticles (LNPs) have attracted extensive attention in tumor immunotherapy. Targeting immune cells in cancer therapy has become a strategy of great research interest. mRNA vaccines are a potential choice for tumor immunotherapy, due to their ability to directly encode antigen proteins and stimulate a strong immune response. However, the mode of delivery and lack of stability of mRNA are key issues limiting its application. LNPs are an excellent mRNA delivery carrier, and their structural stability and biocompatibility make them an effective means for delivering mRNA to specific targets. This study summarizes the research progress in LNP delivery carrier-assisted targeted controlled release mRNA vaccines in tumor immunity. The role of LNPs in improving mRNA stability, immunogenicity, and targeting is discussed. This review aims to systematically summarize the latest research progress in LNP delivery carrier-assisted targeted controlled release mRNA vaccines in tumor immunity to provide new ideas and strategies for tumor immunotherapy, as well as to provide more effective treatment plans for patients.

## 1. Introduction

In the field of cancer treatment, the continuous progress of medical science and technology has ushered in unprecedented changes [1,2,3,4]. As an innovative therapeutic method, tumor immunotherapy has shown great potential in cancer treatment [5]. Compared with traditional treatments, immunotherapy activates and enhances the body’s own immune system to achieve precise effects on tumors, bringing new hope and possibilities for tumor patients [6,7,8,9]. However, although tumor immunotherapy has seen remarkable progress in recent years, its application still faces challenges and limitations [10]. One of the main issues is understanding how to improve the effectiveness and specificity of treatment to maximize the inhibition of tumor growth, spread, and recurrence [11,12,13,14,15,16,17,18]. Compared with traditional vaccines, mRNA vaccines have the advantages of fast preparation, strong customization, and of having no need to use live viruses [19]. With the rise in tumor immunotherapy, researchers have been seeking innovative ways to improve the effectiveness and specificity of treatments to better address tumor challenges. mRNA vaccines have attracted much attention as a potential tumor therapy [20]. Their principle is to guide the body’s cells to synthesize specific antigen proteins encoded by mRNA sequences; the immune system then produces an immune response against the tumor antigens [21,22,23,24,25,26]. However, the clinical use of mRNA vaccines is limited by the challenges in delivery and their lack of stability. In this context, lipid nanoparticles (LNPs), which are nanomaterials, have become key to solving the problem of mRNA vaccine delivery [27,28,29,30]. The LNP structure is made up of lipid layers that wrap the mRNA and protect it from degradation. Through specific surface modification and construction schemes, LNPs can achieve the targeted delivery of mRNA and enhance its enrichment in specific cells or tumor tissues, thereby improving its therapeutic effect [31,32,33,34,35]. The construction scheme of LNPs involves many factors, including selecting the lipid composition, regulating its particle size and surface properties, and optimizing the nucleic acid encapsulation rate [36,37,38,39,40]. For example, the stability and targeting of LNPs can be adjusted by rationally designing different types of lipid components. Optimizing the nucleic acid encapsulation rate can improve the delivery efficiency and bioavailability of mRNA vaccines [41]. In addition, surface modifications can enhance the specific recognition and cellular uptake of LNPs by tumor cells using targeted ligands or polymer functionalization. Preventive vaccines are composed of the following types of nanoparticles: Lipid nanoparticles: These consist of lipid bilayers that can be used to enclose mRNA or protein antigens of pathogens. This type of nanoparticle is widely used in mRNA vaccines (COVID-19 vaccines). Protein nanoparticles: The protein antigen surface of a pathogen is fixed to a nanoparticle to enhance the immune system’s response to the antigen. These nanoparticles can be made from a variety of materials, such as polymers, metals, or other biocompatible materials. Polymer nanoparticles: These include natural or synthetic polymers that can be used to carry antigens or provide appropriate structural support. Virus-like particles: These are nanoparticles that mimic the structure of viruses and do not contain viral nucleic acids, and VLPs can induce the immune system to produce an immune response similar to that of real viruses.

However, there are some challenges in the clinical application of mRNA vaccines, namely their delivery and lack of stability. In recent studies [42,43,44,45], lipid nanoparticles (LNPs) have emerged as an effective mRNA delivery tool. LNPs have excellent biocompatibility and delivery efficiency, can be used as carriers of mRNA vaccines to improve their stability and enhance their targeting, and have shown broad application prospects in tumor immunotherapy.

This review explores the molecular mechanism of LNPs in mRNA vaccine delivery in detail, providing theoretical guidance for the further optimization of LNP design and construction. This information will enhance their effectiveness and safety in tumor immunotherapy and enhance our understanding of the targeted delivery and controlled release mechanism of LNPs, which is helpful in solving the challenges of applying mRNA vaccines in tumor immunotherapy. This study provides a scientific basis for developing more accurate and efficient tumor treatment strategies.

## 2. mRNA Vaccines and Tumor Immunity

### 2.1. Principles and Characteristics of mRNA Vaccines in Tumor Immunotherapy

#### 2.1.1. The Basic Working Principle of mRNA Vaccines

As an innovative tumor therapy, mRNA vaccines work by delivering specific mRNA sequences to stimulate the body’s immune system and induce an antigenic immune response against tumors [46,47,48,49,50]. The vaccine carries mRNA-encoding tumor-specific antigens that, once injected into the body, are taken up by target cells (such as dendritic cells) and translated into antigenic proteins [51]. These proteins are recognized as exogenous within the cell by the innate immune system and activate antigen-presenting cells (APCs), such as dendritic cells. The APCs present these antigens to T cells and stimulate the T cells to produce a specific immune response [52,53,54,55,56]. The activated T cells will then locate and attack tumor cells that have this specific antigen, enabling targeted tumor immunotherapy.

**Figure 1:**
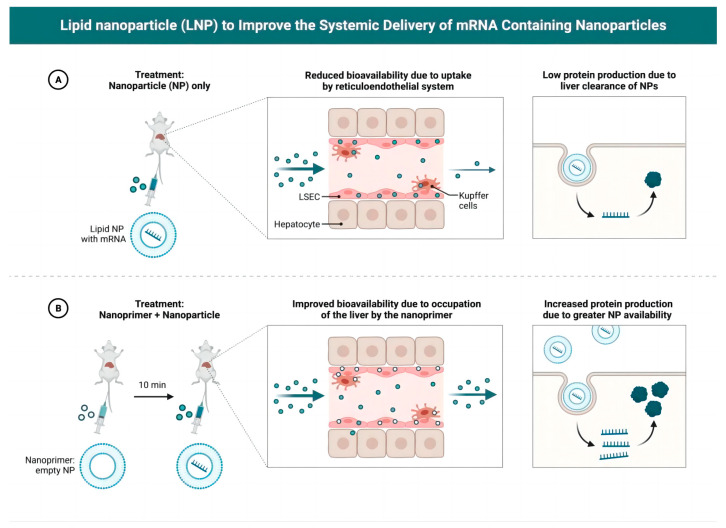
Lipid nanoparticles (LNPs) to improve the systemic delivery of mRNA-containing nanoparticles. (**A**) Treatment with nanoparticle (NP) only; (**B**) treatment with nanoprimer + nanoparticle.

Lipid nanoparticles (LNPs), as nanoparticle carriers containing mRNA, play an important role in whole-body transport [57,58,59,60]. Their superior biocompatibility and efficient intracellular release mechanism make them an ideal drug delivery tool [61,62,63]. LNPs can effectively protect mRNA, improve its stability, and release mRNA inside cells to promote absorption by the target cells (Figure 1).

#### 2.1.2. Characteristics and Advantages of mRNA Vaccines

mRNA vaccines have unique characteristics and advantages in comparison to traditional vaccines [64,65,66]. Their preparation is fast; using modern biotechnology, only the corresponding mRNA sequence is designed, based on the tumor antigen sequence, and there is no need to culture an active virus or prepare a large number of proteins. mRNA vaccines can be highly personalized and can be quickly adjusted to the specific needs of different tumor types or individuals, opening up the possibility of personalized treatment [67,68,69,70]. In addition, because mRNA vaccines can encode specific tumor antigens, they have the potential to target specific tumor antigens, which is expected to provide customized immunotherapy for different tumor types [71,72,73,74]. In addition, mRNA vaccine preparation is relatively simple, reducing the complexity of traditional vaccine production and improving their production efficiency (Table 1).

### 2.2. Current Status and Challenges of mRNA Vaccines in Tumor Therapy

#### 2.2.1. Existing Clinical Application Cases of mRNA Vaccines

At present, several mRNA vaccines have been clinically tested in the field of tumor therapy [75]. For example, some personalized mRNA vaccines targeting specific tumor antigens have shown some clinical efficacy, prompting the body to produce an immune response against the tumor antigen. Some clinical trials [76,77,78,79,80] have shown that these mRNA vaccines show some therapeutic potential in some tumor types and can stimulate the body’s immune system and inhibit tumor growth and spread. However, despite some progress, mRNA vaccines still face some challenges in clinical application [81]. These include stability issues, side effects, and the challenges of generality in different tumor types [82,83,84]. In addition, the results of some clinical trials have not fully confirmed their efficacy and safety, and further large-scale studies and clinical validations are needed [85]. These challenges limit the widespread use of mRNA vaccines in cancer therapy, and further research is needed to improve their efficacy and reliability for clinical use.

#### 2.2.2. The Challenges of mRNA Vaccines

As an emerging cancer therapy, mRNA vaccines face multiple challenges [86,87,88]. The stability of mRNA vaccines is a major concern. Because mRNA is easily degraded, its stability in the body is challenged, potentially leading to the degradation and invalidation of vaccines. Therefore, enhancing the stability of mRNA vaccines and prolonging their existence in vivo have become urgent problems to be solved [89]. Immune response regulation is also one of the challenges faced by mRNA vaccines in tumor therapy [90]. The overactivation of the immune system can lead to adverse reactions, such as immune-related toxicity and immunoreactive side effects [91]. Therefore, balancing and regulating the response of the immune system, in order to ensure that the vaccine does not trigger inappropriate inflammation or autoimmune damage when inducing immunity, are key issues in the application of mRNA vaccines. In addition, the versatility of mRNA vaccines across different tumor types and individuals is also a challenge [92,93,94,95]. Due to tumor heterogeneity and individual patient differences, it is difficult for a single mRNA vaccine to cover all tumor types. Therefore, it is necessary to develop more widely applicable and scalable mRNA vaccines to meet the therapeutic needs of patients with different tumors. In summary, mRNA vaccines face many challenges in tumor therapy, such as stability, immune response regulation, and versatility.

### 2.3. Tumor Immune Mechanism Induced by mRNA Vaccines

#### 2.3.1. Immunogenicity and Immune Memory

**Figure 2:**
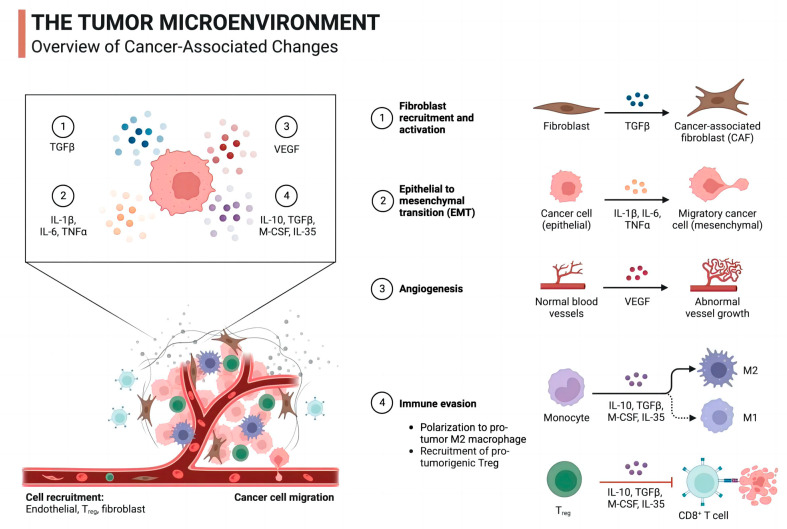
Overview of cancer-associated changes in the tumor microenvironment.

mRNA vaccines activate the body’s immune system by delivering specific mRNA sequences and inducing host cells to synthesize specific tumor antigen proteins [96,97,98]. These antigenic proteins are presented to T cells by antigen-presenting cells, triggering specific immune responses and promoting the activation and proliferation of CD8+ T cells and CD4+ T cells [99]. mRNA vaccines also contribute to the formation of immune memory, allowing the body to remember and recognize specific tumor antigens in the long term, thereby rapidly generating a specific immune response when exposed to the same antigen again (Figure 2).

#### 2.3.2. Immune Cells and Tumor Antigens

mRNA vaccines play an important role in tumor therapy by mobilizing immune cells to recognize and attack tumor-specific antigens [100]. These vaccines work by delivering mRNA sequences encoding tumor-specific antigens, driving antigen expression within host cells and promoting immune system activation. Dendritic cells are key cells in the immune system that are able to take up exogenous antigens and present them to T cells to initiate specific immune responses [101,102,103,104]. mRNA vaccines activate dendritic cells to take up and present tumor-specific antigens, triggering the activation and proliferation of T cells [105]. CD8+ T cells play a key role in this process. They are activated and transformed into killer effector cells that seek out and attack tumor cells that express tumor-specific antigens. On the other hand, CD4+ T helper cells provide auxiliary support, promote the activation and proliferation of CD8+ T cells, and strengthen the immune response [106,107,108,109,110]. In addition to T cells, NK cells also play an important role [111]. mRNA vaccines promote the activation of NK cells, which are able to directly recognize and kill tumor cells expressing tumor antigens, enhancing the immune attack against tumors.

mRNA vaccines can induce the expression of tumor-specific antigens by activating dendritic cells and triggering the activation and proliferation of CD8+ T cells, CD4+ T cells, and NK cells to achieve specific immune attacks against tumors [112,113,114]. An in-depth understanding of this mechanism could help optimize the design of mRNA vaccines and improve their efficacy and safety in tumor immunotherapy.

### 2.4. Development and Future Prospects of mRNA Vaccines in Tumor Immunotherapy

As cutting-edge tumor therapies, mRNA vaccines have shown broad development prospects [115]. In the future, mRNA vaccines are expected to play an important role in tumor treatment, especially in personalized treatment. Their flexibility and customizability enable them to be precisely designed for specific tumor antigens, providing customized treatment options for all types of tumors [116,117,118]. mRNA vaccines are expected to show potential advantages in preventing recurrence, treating metastatic tumors, and assisting other therapeutic methods. In addition, mRNA vaccines may become an important part of tumor immunotherapy in the future, combining with immune checkpoint inhibitors or other immunotherapies to form a diversified tumor treatment regimen.

However, mRNA vaccines still face many challenges in tumor immunotherapy [119], such as in improving their stability, enhancing the specificity and persistence of the immune response, and avoiding immune-related adverse reactions [120,121,122]. The key to addressing these challenges lies in further in-depth research into the design and delivery of mRNA vaccines to enhance their stability and effectiveness in vivo [123,124,125,126]. In addition, the combination of new nanotechnology, biomaterials, and cutting-edge technologies, such as gene editing, is expected to provide more effective solutions and provide more reliable support for developing the application of mRNA vaccines in tumor therapy.

## 3. The Role of Lipid Nanoparticles (LNPs) in mRNA Vaccine Delivery

### 3.1. Structure and Characteristics of LNPs

**Figure 3:**
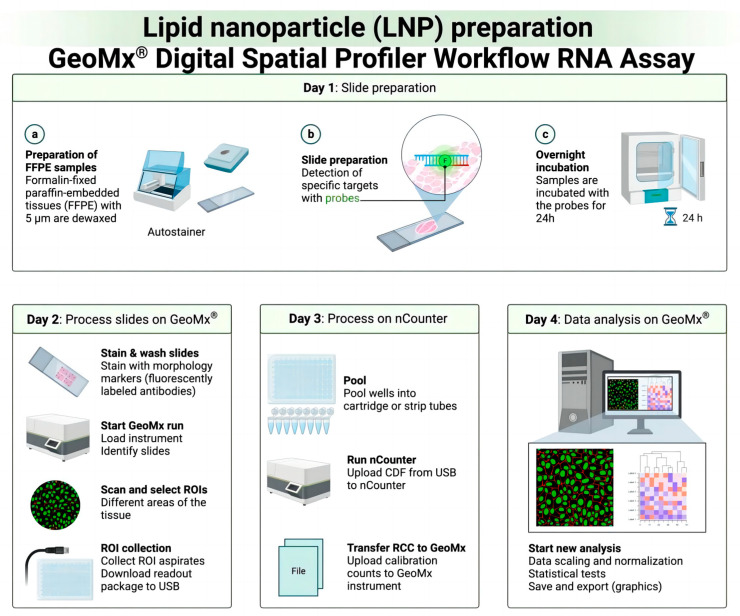
Sequencing technique of the GeoMx™ spatial analysis of RNA in FFPE tissue samples to analyze lipid nanoparticles (LNPs).

As carriers of mRNA vaccines, lipid nanoparticles (LNPs) play an important role in mRNA delivery [127,128,129,130]. LNPs are usually composed of hydrophobic lipids, cholesterol, PEG-modified lipids, and ionic surfactants, and they come in different nanomedicine carrier types with different applications, as shown in Table 2. These components form a nanoscale structure whose core is a lipid double layer made of hydrophobic lipids that envelop the mRNA vaccine [131,132,133,134]. This structure gives LNPs excellent biocompatibility and stability, helping to protect the mRNA from degradation. In addition, the surfaces of LNPs are often modified with PEG, which can improve their blood circulation time and reduce the chance of them being cleared by the immune system. LNPs have multiple advantages in RNA vaccine delivery [135,136,137]. Their lipid bilayer structure can effectively encapsulate mRNA vaccines and protect them from degradation by the external environment, which helps to improve the stability of the mRNA. LNPs can improve the biological distribution of mRNA in the body, enhance its cell uptake efficiency, and promote its delivery to target cells, thus enhancing the effectiveness of mRNA vaccines [138,139,140]. With GeoMx™ spatial analysis, scientists were able to delve deeper into the RNA needed to build lipid nanoparticles (LNPs) to more fully understand their composition and properties (Figure 3).

### 3.2. Delivery Mechanism of LNPs as mRNA Vaccine Carriers

**Figure 4:**
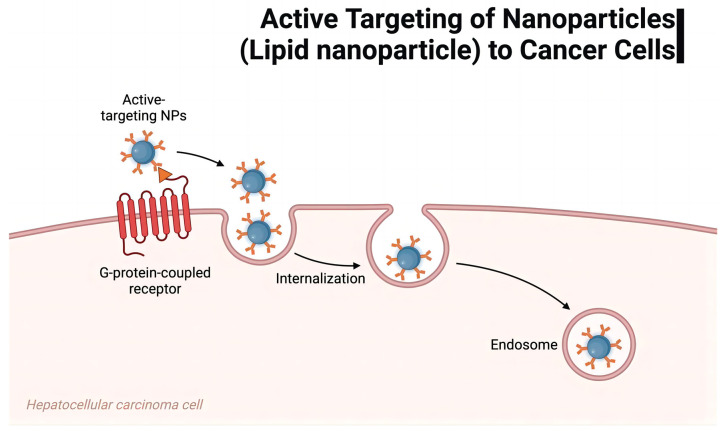
Active targeting of lipid nanoparticles (LNPs) to cancer cells.

As carriers of mRNA vaccines, lipid nanoparticles (LNPs) play an important role in tumor therapy [141]. Their delivery mechanism mainly manifests in two aspects: targeted delivery and controlled release. LNPs achieve the targeted delivery of mRNA vaccines through their special structural and chemical properties [142,143,144]. The lipid bilayer structure of LNPs enables them to encapsulate mRNA vaccines, forming stable nanoparticles that help protect the mRNA from degradation [145]. In addition, the LNP surface can be targeted by changing the lipid composition and surface modifications [146]. Tumor-specific surface markers can improve the affinity of LNPs to tumor tissues, promote the enrichment of LNP carriers and their supported mRNA vaccines in tumor cells, and reduce their impact on healthy tissues. LNPs have the characteristic of controlled release, which helps to improve the effect of mRNA vaccines [147,148,149,150]. Researchers can achieve the controlled release of mRNA by regulating the lipid composition and structure of LNPs so that the mRNA vaccine can be maintained in the body for a longer time and enhance its therapeutic effect (Figure 4). In addition, LNPs can also promote the intracellular uptake of mRNA so that mRNA vaccines can enter the cell more effectively and initiate the immune response to improve the specific attack ability of tumor cells.

**Figure 5:**
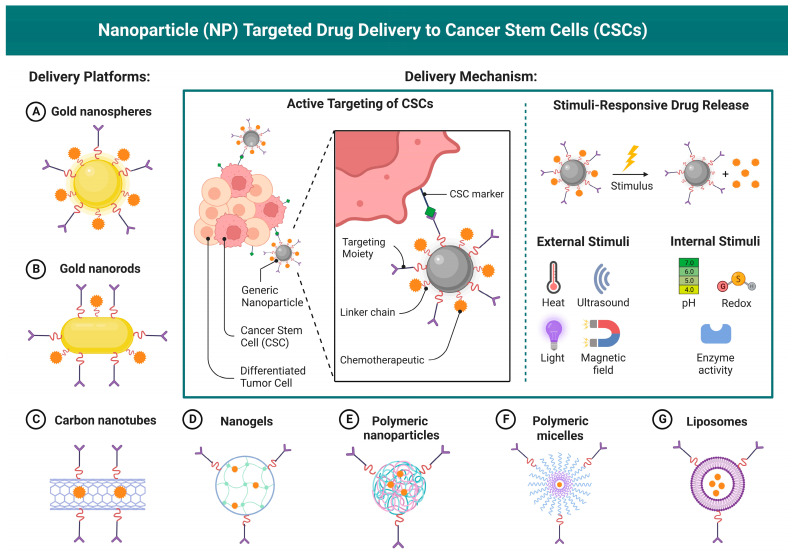
Lipid nanoparticle (LNP)-targeted drug delivery to cancer stem cells.

As the carrier of mRNA vaccines, lipid nanoparticles (LNPs) can improve the effectiveness of mRNA vaccines in tumor therapy through targeted delivery and controlled release mechanisms [151,152,153]. Their targeting and controlled release properties make them a potential tumor therapeutic delivery tool, which is expected to lead to more accurate and effective treatment strategies for tumor immunotherapy (Figure 5).

## 4. Application of LNP-Assisted mRNA Vaccines in Tumor Immunotherapy

### 4.1. Progress of Experimental Research

In the field of tumor therapy, the application of LNP-assisted mRNA vaccines has aroused extensive research interest [154]. In past studies [155,156,157,158,159,160], researchers have achieved a series of encouraging results by using LNP carriers to deliver mRNA vaccines to tumor models (such as liver cancer). Some studies [161,162,163,164] have shown that LNP carriers can effectively deliver mRNA vaccines and stimulate tumor antigen-specific immune responses in liver tumor mouse models. For example, some mRNA vaccines targeting tumor-specific antigens delivered through LNP carriers can induce high levels of specific antibodies and cellular immune responses, inhibit tumor growth, and prolong the survival time of mice [165]. In addition, LNP-assisted mRNA vaccines have also been shown to activate CD8+ T cells and enhance immune cell recognition and attacks on tumors, playing an important role in tumor inhibition. Some studies [166,167,168,169,170] have pointed out that LNP carriers can help improve the stability and intracellular uptake efficiency of mRNA vaccines, thus enhancing the biological activity and persistence of mRNA vaccines. These findings provide strong support and evidence for the application of LNP-assisted mRNA vaccines in tumor therapy [171].

**Figure 6:**
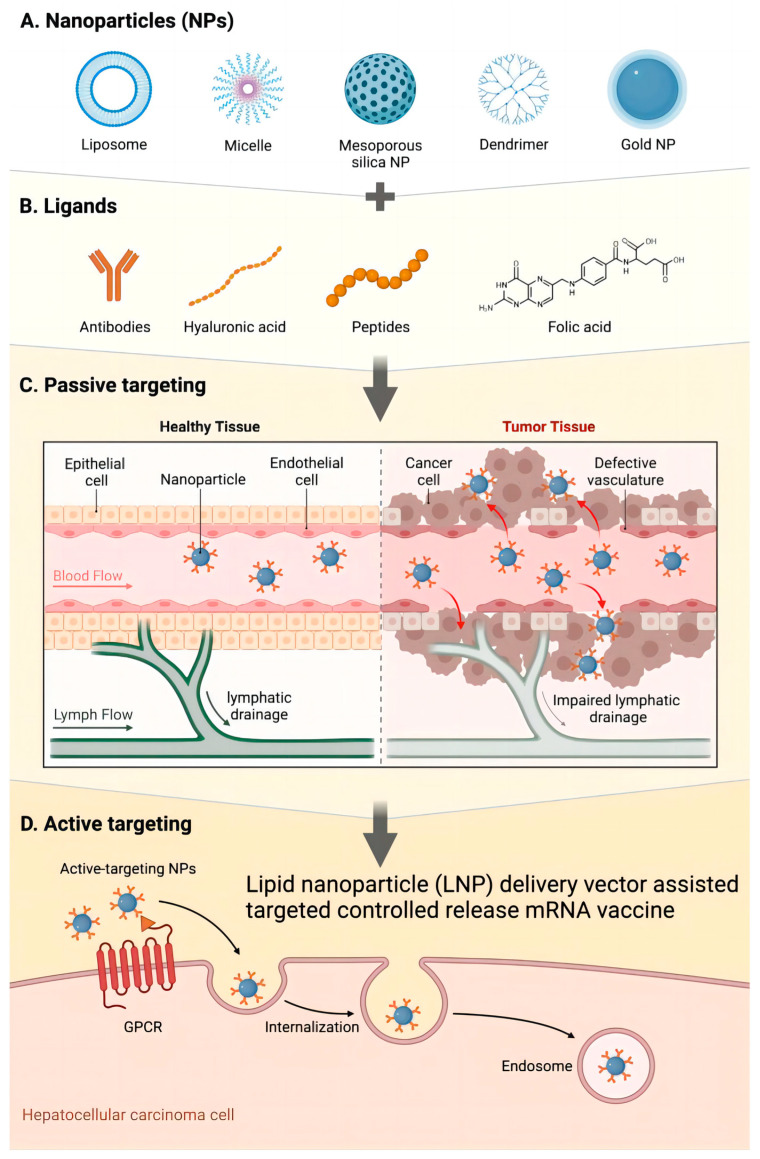
Lipid nanoparticle (LNP) drug delivery systems target liver cancer. (**A**) Nanoparticles (NPs); (**B**) ligands; (**C**) passive targeting; (**D**) active targeting.

However, despite these positive research advances, there remain some challenges and necessary directions for future research [172]. LNP carriers’ biological distribution, stability, and interaction with the immune system still need to be further studied to improve their delivery efficiency and reduce any potential toxic effects [173,174,175]. At the same time, more preclinical studies and clinical trials will help to fully evaluate the potential use of LNP-assisted mRNA vaccines in tumor immunotherapy, as well as their safety and efficacy (Figure 6).

### 4.2. Other Potential Application Areas

LNP auxiliary mRNA vaccines are not limited to single applications in the field of tumor immunotherapy; they also show broad prospects for combined applications, especially in combination with other immunotherapies [176,177,178,179,180]. This combined treatment strategy is expected to improve the effectiveness of tumor therapy, enhance the immune response, and overcome the limitations of a single treatment approach [181,182,183,184]. One potential application is the combination of LNP-assisted mRNA vaccines with immune checkpoint inhibitors. Immune checkpoint inhibitors activate the body’s immune system to fight tumors by removing the immunosuppression of tumor cells on T cells [185,186,187,188]. LNP auxiliary mRNA vaccines can stimulate and enhance the immune response to tumor-specific antigens [189]. The combined application of the two is expected to be complementary, could improve the effect of tumor immunotherapy, and may expand their application scope [190]. In addition, the combination of LNP-assisted mRNA vaccines with other immunotherapies, such as CAR T cell therapy or tumor vaccines, is also attracting attention [191,192,193,194]. This combined application can work synergistically to enhance multiple attacks on tumors. For example, mRNA vaccines can induce the body to produce specific antibodies and T cell immune responses, while CAR T cell therapy works by modifying T cells to directly recognize and attack tumor cells; a combination of the two may achieve more comprehensive and long-lasting tumor treatment effects. However, these combination treatment strategies require more in-depth research to address a number of challenges, including the optimization of treatment protocols, the management of side effects, and the long-term monitoring of treatment effects [195,196,197,198]. In addition, the specific mechanisms and interactions of combination therapy also need to be clarified in additional experimental and clinical studies [199].

LNP auxiliary mRNA vaccines have great potential in combination with other immunotherapies, which can provide more comprehensive and effective treatment strategies for tumor immunotherapy and can provide more treatment options for patients.

## 5. Future Prospects and Challenges

As carriers of mRNA vaccines, LNPs show great potential in tumor immunotherapy, but they still face a series of challenges and development directions [200,201,202,203,204,205]. The future development trend of LNP carriers in tumor immunotherapy may see a focus on improving their delivery efficiency and accuracy [206,207,208,209,210]. This would include further improving the design of LNPs and optimizing their distribution and stability in vivo in order to improve the delivery efficiency and antitumor effect of mRNA vaccines [211]. At the same time, according to different tumor types and individual patient differences, the development of personalized and customized LNP carriers and mRNA vaccine programs is also an important direction for future development [212,213,214,215,216,217,218]. LNP research in tumor immunotherapy will also focus more on safety and on the management of side effects. With the promotion of LNPs in clinical applications, it is necessary to have more in-depth understandings of their metabolic dynamics and toxic reactions in the body, of engaging in the timely detection and remediation of potential safety risks, and of ensuring the safety and controllability of the treatment [219,220,221,222]. In addition, in the future, LNP carriers may be combined with emerging technologies, such as nanotechnology and gene editing, in order to explore a variety of new therapeutic strategies [223,224,225,226]. For example, novel nanomaterials or carrier technologies can be combined to optimize LNP delivery characteristics [227,228,229,230]. Alternatively, gene editing technology and LNP carriers can be combined to achieve the accurate editing and regulation of tumor genes, bringing about more possibilities in tumor treatment [231,232,233,234,235,236].

However, there are still some challenges in the future development of LNP carriers in tumor immunotherapy [237,238,239,240]. This includes improving their delivery efficiency and specificity, overcoming immune-related side effects, exploring more effective targeting strategies, and reducing costs to improve production processes. Addressing these challenges requires interdisciplinary collaboration, the integration of technologies and resources, strengthening of basic research and clinical trials, and the continuous improvement of regulatory policies in order to drive continued innovation in the development of LNP carriers in the field of tumor immunotherapy [241,242,243,244,245,246,247,248].

LNPs have potential as carriers for mRNA vaccines in tumor therapy, but there are still some challenges that need to be overcome to achieve their widespread application [249]. One of the challenges is the stability and immunogenicity of LNPs in vivo [250,251,252]. LNPs may suffer from protein adsorption and micellar rupture in blood circulation, limiting their ability to effectively deliver mRNA vaccines. One solution may be to improve the surface modifications of LNPs, using a variety of modifications (e.g., PEG-ification) to improve their stability and blood circulation time and to reduce immune responses [253,254,255]. Another challenge is the liver enrichment of LNPs. LNPs tend to be concentrated in the liver rather than tumor tissue, which limits their precise delivery to tumors [256,257,258,259,260]. In response to this challenge, we can explore improving the targeting of LNPs, designing specific targeting ligands or functionalized molecules, and making them more inclined to be enriched in tumor tissues in order to improve the therapeutic effect [261]. Lipid nanoparticles (LNP) are a common vaccine delivery system consisting of different lipid components. Typical LNPs include neutral lipids, charged lipids, and pegylated lipids. Neutral lipids are usually composed of phospholipids, such as phosphatidylcholine, which provide structural support for nanoparticles. Charged lipids, such as choline salts, give LNP a charge that helps stabilize and improve the encapsulation efficiency of nucleic acids. Pegylated lipids are used to coat the surface of the nanoparticles, forming a protective layer to slow down the clearance of LNPs by the immune system. These components work together to achieve the efficient delivery of pathogen antigens and trigger immune system responses. By carefully designing these chemical structures, LNPs not only improve the stability and delivery efficiency of the vaccine but also reduce the immune response, providing strong support for vaccine research and development.

In addition, the LNP preparation process, production cost, and scale of production are also challenges [262,263,264,265]. To solve these problems, it is necessary to optimize the preparation process, increase the yield, reduce the cost, and promote large-scale production. To address these challenges, interdisciplinary collaboration is essential [266]. Combining expertise in biomedical science, nanotechnology, materials science, and other fields strengthens research cooperation and helps to jointly overcome technical problems [267]. In addition, the guidance and norms of regulatory policies should be strengthened to ensure the safety and effectiveness of LNPs in clinical applications. Notably, some of the vaccine components have been found in the milk of lactating mothers. This finding raises safety concerns that require in-depth discussion and research. In particular, in the presence of vaccine components in the milk of lactating mothers, we need to understand the source, magnitude, and potential impacts these vaccine components have on infant health. When discussing safety, we must consider the importance of breastfeeding in the health of the baby and evaluate it in the context of ensuring their safety [268]. This will help ensure the metabolizing and excretion of the vaccine in lactating mothers and provide a more comprehensive safety assessment for the health of both the mother and child.

In summary, overcoming the challenges faced by LNPs as mRNA vaccine carriers in tumor therapy requires multifaceted efforts and innovation. By continuously improving the stability, targeting, and production technology of LNPs, combined with reasonable research and development strategies, it is believed that LNPs will have broader application prospects in tumor therapy.

## 6. Conclusions

This study summarized the key role of LNPs as mRNA vaccine carriers in tumor immunotherapy. LNPs can promote the targeted delivery and controlled release of mRNA vaccines, stimulate the immune response, and fight against tumors. The advantages of mRNA vaccines are their rapid preparation, personalized customization, potential for specific tumor antigens, etc. They are expected to become an innovative means of tumor treatment; LNP-assisted mRNA vaccines have achieved encouraging therapeutic effects in tumor models.

In the future, the development prospects of LNP-assisted mRNA vaccines in tumor therapy are broad. The potential for personalized treatment and the application of combined immunotherapy will become an important research direction. However, challenges such as stability, targeting, and the advancing of preclinical and clinical studies still need to be addressed. Further studying LNP structure optimization, targeting strategies, and multidisciplinary cooperation are suggested approaches for improving the effective application of LNP-assisted mRNA vaccines in tumor therapy and promoting their clinical transformation.

## Figures and Tables

**Table 1:** Analysis of the application of mRNA vaccine types and bionanomaterial carriers.

mRNA Vaccine Type	mRNA Vaccine Carrier Properties	Related Research	Specific Disease Applications	Types of Bionanomaterials Used with mRNA Vaccines
Lipid Nanoparticles [64] (LNP)	High encapsulation, intracellular delivery	Pfizer-BioNTech, Moderna	COVID-19	Liposomes, Polymeric Nanoparticles
Polymeric Nanoparticles [65]	Tunable release, stability	CureVac	COVID-19, Vaccine Development	Polymers, Liposomes
Protein–Polymer Nanocomplexes [66]	Targeted, stability	Arcturus Therapeutics	COVID-19, Vaccine Development	Proteins, Polymers
Lipid–Protein Complexes [67,68,69]	Efficient transfection, mRNA protection	Acuitas Therapeutics	COVID-19, Other Vaccines	Lipids, Proteins
Lipid–Peptide Complexes [70]	Specific targeting, enhanced immunity	Moderna	COVID-19	Lipids, Peptides
Nano-Peptide Particles [71]	Antigen presentation, immune activation	Stanford Research	COVID-19, Cancer Vaccines	Peptides
Magnetic Nanoparticles [72]	Imaging-guided, vaccine delivery	Under Research	Cancer, Vaccine Development	Iron Oxide Magnetic Nanoparticles
Metal–Organic Frameworks (MOFs) [73]	High drug loading, controlled release	Under Research	Vaccine Development	MOFs, mRNA Vaccines
Carbon-Based Nanomaterials [74]	Biocompatibility, delivery efficiency	Under Research	Cancer Immunotherapy	Carbon Nanotubes, Graphene Oxide
Gold Nanoparticles [75]	Efficient transport, immune activation	Under Research	Cancer, Vaccine Development	Gold Nanoparticles, mRNA Vaccines

**Table 2:** Induction and analysis of nanomedicine carrier types and applications.

Nanoparticle Carrier Type	Nanomaterial Properties	Related Research	Targeted Tumor	Types of Nanomedicine
Liposomes [127]	Lipid bilayer structure, high encapsulation ability	Doxil, Onivyde	Ovarian cancer, Pancreatic cancer	Chemotherapeutic drug delivery
Polymeric Nanoparticles [128]	Tunable release properties	Abraxane, Genexol-PM	Breast cancer, Gastric cancer	Chemotherapeutic drug delivery
Gold Nanoparticles [129]	Biocompatibility, surface-enhanced Raman scattering	-	Lung cancer, Breast cancer	Tumor photothermal therapy
Iron Oxide Magnetic Nanoparticles [130]	Magnetic properties, imaging functionality	Ferumoxytol	Brain tumors, Breast cancer	Magnetic resonance imaging
Metal–Organic Frameworks (MOFs) [131]	High drug-loading capacity, controlled release	-	Lung cancer, Colorectal cancer	Drug delivery, Imaging
Graphene Oxide [132]	Large surface area, drug-loading capability	-	Lung cancer, Breast cancer	Drug delivery
Carbon Nanotubes [133]	High drug-loading capacity, biocompatibility	-	Lung cancer, Breast cancer	Drug delivery, Photothermal therapy
Protein Nanoparticles [134]	Biocompatibility, specific targeting	Abraxane	Pancreatic cancer, Ovarian cancer	Protein drug delivery
Lipid Nanoparticles [135]	Biocompatibility, high drug-loading capacity	Pfizer-BioNTech mRNA vaccine	Breast cancer, Colorectal cancer	mRNA vaccines
Iron Oxide Nanoparticles [136]	Magnetic properties, imaging functionality	-	Liver cancer, Breast cancer	Magnetic resonance imaging
PLGA Nanoparticles [137]	Biodegradability, controlled release	-	Lung cancer, Breast cancer	Drug delivery
Protein–Polymer Nanocomplexes [138,139,140]	Targeted, biocompatible	-	Gastric cancer, Colorectal cancer	Protein drug delivery
Phospholipid Nanoparticles [141]	Biocompatibility, stability	-	Gastric cancer, Liver cancer	Drug delivery
Silica Nanoparticles [142]	Tunable morphology, drug-loading capability	-	Liver cancer, Breast cancer	Drug delivery
Polymer Micelles [143]	High drug-loading capacity, solubility	-	Lung cancer, Pancreatic cancer	Chemotherapeutic drug delivery
Nanoemulsions [144]	Drug-carrying capacity, stability	-	Pancreatic cancer, Colorectal cancer	Drug delivery, Treatment

## Data Availability

Not applicable.

## References

[1] Bevers S., Kooijmans S.A.A., Van de Velde E., Evers M.J.W., Seghers S., Gitz-Francois J.J.J.M., van Kronenburg N.C.H., Fens M.H.A.M., Mastrobattista E., Hassler L. (2022). mRNA-LNP vaccines tuned for systemic immunization induce strong antitumor immunity by engaging splenic immune cells. Mol. Ther..

[2] Yeh T.F., Lin C., Sung H.C. (2023). A review of technological developments in lipid nanoparticle application for mRNA vaccination. Hum. Vaccines Immunother..

[3] Sittplangkoon C., Alameh M.G., Weissman D., Lin P.J.C., Tam Y.K., Prompetchara E., Palaga T. (2022). mRNA vaccine with unmodified uridine induces robust type I interferon-dependent anti-tumor immunity in a melanoma model. Front. Immunol..

[4] Fei Q., Shalosky E.M., Barnes R., Shukla V.C., Xu S., Ballinger M.N., Farkas L., Lee R.J., Ghadiali S.N., Englert J.A. (2023). Macrophage-Targeted Lipid Nanoparticle Delivery of microRNA-146a to Mitigate Hemorrhagic Shock-Induced Acute Respiratory Distress Syndrome. ACS Nano.

[5] Li F., Zhang X.Q., Ho W., Tang M., Li Z., Bu L., Xu X. (2023). mRNA lipid nanoparticle-mediated pyroptosis sensitizes immunologically cold tumors to checkpoint immunotherapy. Nat. Commun..

[6] Liu W., Alameh M.G., Yang J.F., Xu J.R., Lin P.J.C., Tam Y.K., Weissman D., You J. (2022). Lipid Nanoparticles Delivering Constitutively Active STING mRNA to Stimulate Antitumor Immunity. Int. J. Mol. Sci..

[7] Kitte R., Rabel M., Geczy R., Park S., Fricke S., Koehl U., Tretbar U.S. (2023). Lipid nanoparticles outperform electroporation in mRNA-based CAR T cell engineering. Mol. Ther. Methods Clin. Dev..

[8] Jung H.N., Lee S.Y., Lee S., Youn H., Im H.J. (2022). Lipid nanoparticles for delivery of RNA therapeutics: Current status and the role of in vivoimaging. Theranostics.

[9] Golubovskaya V., Sienkiewicz J., Sun J., Zhang S., Huang Y., Zhou H., Harto H., Xu S., Berahovich R., Wu L. (2023). CAR-NK Cells Generated with mRNA-LNPs Kill Tumor Target Cells In Vitro and In Vivo. Int. J. Mol. Sci..

[10] Onuma H., Sato Y., Harashima H. (2023). Lipid nanoparticle-based ribonucleoprotein delivery for in vivo genome editing. J. Control. Release Off. J. Control. Release Soc..

[11] Schoenmaker L., Witzigmann D., Kulkarni J.A., Verbeke R., Kersten G., Jiskoot W., Crommelin D.J.A. (2021). mRNA-lipid nanoparticle COVID-19 vaccines: Structure and stability. Int. J. Pharm..

[12] Billingsley M.M., Hamilton A.G., Mai D., Patel S.K., Swingle K.L., Sheppard N.C., June C.H., Mitchell M.J. (2022). Orthogonal Design of Experiments for Optimization of Lipid Nanoparticles for mRNA Engineering of CAR T Cells. Nano Lett..

[13] Alameh M.G., Tombácz I., Bettini E., Lederer K., Sittplangkoon C., Wilmore J.R., Gaudette B.T., Soliman O.Y., Pine M., Hicks P. (2021). Lipid nanoparticles enhance the efficacy of mRNA and protein subunit vaccines by inducing robust T follicular helper cell and humoral responses. Immunity.

[14] Zhang N.N., Li X.F., Deng Y.Q., Zhao H., Huang Y.J., Yang G., Huang W.J., Gao P., Zhou C., Zhang R.R. (2020). A Thermostable mRNA Vaccine against COVID-19. Cell.

[15] Kon E., Elia U., Peer D. (2022). Principles for designing an optimal mRNA lipid nanoparticle vaccine. Curr. Opin. Biotechnol..

[16] Hassett K.J., Higgins J., Woods A., Levy B., Xia Y., Hsiao C.J., Acosta E., Almarsson Ö., Moore M.J., Brito L.A. (2021). Impact of lipid nanoparticle size on mRNA vaccine immunogenicity. J. Control Release.

[17] Huang J., Yuen D., Mintern J.D., Johnston A.P.R. (2021). Opportunities for innovation: Building on the success of lipid nanoparticle vaccines. Curr. Opin. Colloid. Interface Sci..

[18] Verbeke R., Hogan M.J., Loré K., Pardi N. (2022). Innate immune mechanisms of mRNA vaccines. Immunity.

[19] Muramatsu H., Lam K., Bajusz C., Laczkó D., Karikó K., Schreiner P., Martin A., Lutwyche P., Heyes J., Pardi N. (2022). Lyophilization provides long-term stability for a lipid nanoparticle-formulated, nucleoside-modified mRNA vaccine. Mol. Ther..

[20] Johnson L.T., Zhang D., Zhou K., Lee S.M., Liu S., Dilliard S.A., Farbiak L., Chatterjee S., Lin Y.H., Siegwart D.J. (2022). Lipid Nanoparticle (LNP) Chemistry Can Endow Unique In Vivo RNA Delivery Fates within the Liver That Alter Therapeutic Outcomes in a Cancer Model. Mol. Pharm..

[21] Oude Blenke E., Örnskov E., Schöneich C., Nilsson G.A., Volkin D.B., Mastrobattista E., Almarsson Ö., Crommelin D.J.A. (2023). The Storage and In-Use Stability of mRNA Vaccines and Therapeutics: Not A Cold Case. J. Pharm. Sci..

[22] Wang M.M., Wappelhorst C.N., Jensen E.L., Chi Y.T., Rouse J.C., Zou Q. (2023). Elucidation of lipid nanoparticle surface structure in mRNA vaccines. Sci. Rep..

[23] Korzun T., Moses A.S., Diba P., Sattler A.L., Taratula O.R., Sahay G., Taratula O., Marks D.L. (2023). From Bench to Bedside: Implications of Lipid Nanoparticle Carrier Reactogenicity for Advancing Nucleic Acid Therapeutics. Pharmaceuticals.

[24] Lam K., Schreiner P., Leung A., Stainton P., Reid S., Yaworski E., Lutwyche P., Heyes J. (2023). Optimizing Lipid Nanoparticles for Delivery in Primates. Adv. Mater..

[25] Han X., Gong N., Xue L., Billingsley M.M., El-Mayta R., Shepherd S.J., Alameh M.G., Weissman D., Mitchell M.J. (2023). Ligand-tethered lipid nanoparticles for targeted RNA delivery to treat liver fibrosis. Nat. Commun..

[26] McMahon M., O’Dell G., Tan J., Sárközy A., Vadovics M., Carreño J.M., Puente-Massaguer E., Muramatsu H., Bajusz C., Rijnink W. (2022). Assessment of a quadrivalent nucleoside-modified mRNA vaccine that protects against group 2 influenza viruses. Proc. Natl. Acad. Sci. USA.

[27] Monslow M.A., Elbashir S., Sullivan N.L., Thiriot D.S., Ahl P., Smith J., Miller E., Cook J., Cosmi S., Thoryk E. (2020). Immunogenicity generated by mRNA vaccine encoding VZV gE antigen is comparable to adjuvanted subunit vaccine and better than live attenuated vaccine in nonhuman primates. Vaccine.

[28] Shen Z., Zhang S., Jiang Q., Liu N., Li F., Gao Z., Pan S., Hao W., Deng Q., Liu J. (2023). Lipid nanoparticle-mediated delivery of IL-21-encoding mRNA induces viral clearance in mouse models of hepatitis B virus persistence. J. Med. Virol..

[29] Hoffmann M.A.G., Yang Z., Huey-Tubman K.E., Cohen A.A., Gnanapragasam P.N.P., Nakatomi L.M., Storm K.N., Moon W.J., Lin P.J.C., West A.P. (2023). ESCRT recruitment to SARS-CoV-2 spike induces virus-like particles that improve mRNA vaccines. Cell.

[30] Pardi N., Hogan M.J., Naradikian M.S., Parkhouse K., Cain D.W., Jones L., Moody M.A., Verkerke H.P., Myles A., Willis E. (2018). Nucleoside-modified mRNA vaccines induce potent T follicular helper and germinal center B cell responses. J. Exp. Med..

[31] Laczkó D., Hogan M.J., Toulmin S.A., Hicks P., Lederer K., Gaudette B.T., Castaño D., Amanat F., Muramatsu H., Oguin T.H. (2020). A Single Immunization with Nucleoside-Modified mRNA Vaccines Elicits Strong Cellular and Humoral Immune Responses against SARS-CoV-2 in Mice. Immunity.

[32] Zheng L., Bandara S.R., Tan Z., Leal C. (2020). Lipid nanoparticle topology regulates endosomal escape and delivery of RNA to the cytoplasm. Proc. Natl. Acad. Sci. USA.

[33] Pardi N., Carreño J.M., O’Dell G., Tan J., Bajusz C., Muramatsu H., Rijnink W., Strohmeier S., Loganathan M., Bielak D. (2022). Development of a pentavalent broadly protective nucleoside-modified mRNA vaccine against influenza B viruses. Nat. Commun..

[34] Douka S., Brandenburg L.E., Casadidio C., Walther J., Garcia B.B.M., Spanholtz J., Raimo M., Hennink W.E., Mastrobattista E., Caiazzo M. (2023). Lipid nanoparticle-mediated messenger RNA delivery for ex vivo engineering of natural killer cells. J. Control. Release Off. J. Control. Release Soc..

[35] Pilkington E.H., Suys E.J.A., Trevaskis N.L., Wheatley A.K., Zukancic D., Algarni A., Al-Wassiti H., Davis T.P., Pouton C.W., Kent S.J. (2021). From influenza to COVID-19: Lipid nanoparticle mRNA vaccines at the frontiers of infectious diseases. Acta Biomater..

[36] Haley R.M., Chan A., Billingsley M.M., Gong N., Padilla M.S., Kim E.H., Wang H., Yin D., Wangensteen K.J., Tsourkas A. (2023). Lipid Nanoparticle Delivery of Small Proteins for Potent In Vivo RAS Inhibition. ACS Appl. Mater. Interfaces.

[37] Pardi N., Hogan M.J., Pelc R.S., Muramatsu H., Andersen H., DeMaso C.R., Dowd K.A., Sutherland L.L., Scearce R.M., Parks R. (2017). Zika virus protection by a single low-dose nucleoside-modified mRNA vaccination. Nature.

[38] Liang Q., Wang Y., Zhang S., Sun J., Sun W., Li J., Liu Y., Li M., Cheng L., Jiang Y. (2022). RBD trimer mRNA vaccine elicits broad and protective immune responses against SARS-CoV-2 variants. iScience.

[39] Jaradat E., Weaver E., Meziane A., Lamprou D.A. (2022). Microfluidic paclitaxel-loaded lipid nanoparticle formulations for chemotherapy. Int. J. Pharm..

[40] Liu T., Tian Y., Zheng A., Cui C. (2022). Design Strategies for and Stability of mRNA-Lipid Nanoparticle COVID-19 Vaccines. Polymers.

[41] Suzuki Y., Ishihara H. (2021). Difference in the lipid nanoparticle technology employed in three approved siRNA (Patisiran) and mRNA (COVID-19 vaccine) drugs. Drug Metab. Pharmacokinet..

[42] Han X., Alameh M.G., Butowska K., Knox J.J., Lundgreen K., Ghattas M., Gong N., Xue L., Xu Y., Lavertu M. (2023). Adjuvant lipidoid-substituted lipid nanoparticles augment the immunogenicity of SARS-CoV-2 mRNA vaccines. Nat. Nanotechnol..

[43] Lederer K., Castaño D., Gómez Atria D., Oguin T.H., Wang S., Manzoni T.B., Muramatsu H., Hogan M.J., Amanat F., Cherubin P. (2020). SARS-CoV-2 mRNA Vaccines Foster Potent Antigen-Specific Germinal Center Responses Associated with Neutralizing Antibody Generation. Immunity.

[44] Zong Y., Lin Y., Wei T., Cheng Q. (2023). Lipid Nanoparticle (LNP) Enables mRNA Delivery for Cancer Therapy. Adv. Mater..

[45] Xu Y., Fourniols T., Labrak Y., Préat V., Beloqui A., Des Rieux A. (2022). Surface Modification of Lipid-Based Nanoparticles. ACS Nano.

[46] Hamilton A.G., Swingle K.L., Joseph R.A., Mai D., Gong N., Billingsley M.M., Alameh M.G., Weissman D., Sheppard N.C., June C.H. (2023). Lonizable Lipid Nanoparticles with Integrated Immune Checkpoint Inhibition for mRNA CAR T Cell Engineering. Adv. Healthc. Mater..

[47] Shi D., Toyonaga S., Anderson D.G. (2023). In Vivo RNA Delivery to Hematopoietic Stem and Progenitor Cells via Targeted Lipid Nanoparticles. Nano Lett..

[48] Jansen E.M., Frijlink H.W., Hinrichs W.L., Ruigrok M.J. (2022). Are inhaled mRNA vaccines safe and effective? A review of preclinical studies. Expert. Opin. Drug Deliv..

[49] Zhong Z., Chen Y., Deswarte K., Lauwers H., De Lombaerde E., Cui X., Van Herck S., Ye T., Gontsarik M., Lienenklaus S. (2023). Lipid Nanoparticle Delivery Alters the Adjuvanticity of the TLR9 Agonist CpG by Innate Immune Activation in Lymphoid Tissue. Adv. Healthc. Mater..

[50] Athirasala A., Patel S., Menezes P.P., Kim J., Tahayeri A., Sahay G., Bertassoni L.E. (2022). Matrix stiffness regulates lipid nanoparticle-mRNA delivery in cell-laden hydrogels. Nanomed. Nanotechnol. Biol. Med..

[51] Driscoll D.F. (2023). Lipid nanoparticle-based COVID-19 vaccines: Ensuring pharmaceutical stability, safety, and efficacy. Am. J. Health-Syst. Pharm. AJHP Off. J. Am. Soc. Health-Syst. Pharm..

[52] Lu J., Wei W., He W. (2023). Regulatory perspective for quality evaluation of lipid nanoparticle-based mRNA vaccines in China. Biol. J. Int. Assoc. Biol. Stand..

[53] Deng Y.Q., Zhang N.N., Zhang Y.F., Zhong X., Xu S., Qiu H.Y., Wang T.C., Zhao H., Zhou C., Zu S.L. (2022). Lipid nanoparticle-encapsulated mRNA antibody provides long-term protection against SARS-CoV-2 in mice and hamsters. Cell Res..

[54] Le Saux S., Aubert-Pouëssel A., Mohamed K.E., Martineau P., Guglielmi L., Devoisselle J.M., Legrand P., Chopineau J., Morille M. (2021). Interest of extracellular vesicles in regards to lipid nanoparticle based systems for intracellular protein delivery. Adv. Drug Deliv. Rev..

[55] Kim B., Hosn R.R., Remba T., Yun D., Li N., Abraham W., Melo M.B., Cortes M., Li B., Zhang Y. (2023). Optimization of storage conditions for lipid nanoparticle-formulated self-replicating RNA vaccines. J. Control. Release Off. J. Control. Release Soc..

[56] Huang C., Duan X., Wang J., Tian Q., Ren Y., Chen K., Zhang Z., Li Y., Feng Y., Zhong K. (2023). Lipid Nanoparticle Delivery System for mRNA Encoding B7H3-redirected Bispecific Antibody Displays Potent Antitumor Effects on Malignant Tumors. Adv. Sci..

[57] Ge N., Sun J., Liu Z., Shu J., Yan H., Kou Z., Wei Y., Jin X. (2022). An mRNA vaccine encoding Chikungunya virus E2-E1 protein elicits robust neutralizing antibody responses and CTL immune responses. Virol. Sin..

[58] Khalin I., Adarsh N., Schifferer M., Wehn A., Groschup B., Misgeld T., Klymchenko A., Plesnila N. (2022). Size-Selective Transfer of Lipid Nanoparticle-Based Drug Carriers Across the Blood Brain Barrier via Vascular Occlusions Following Traumatic Brain Injury. Small.

[59] Zimmermann C.M., Baldassi D., Chan K., Adams N.B.P., Neumann A., Porras-Gonzalez D.L., Wei X., Kneidinger N., Stoleriu M.G., Burgstaller G. (2022). Spray drying siRNA-lipid nanoparticles for dry powder pulmonary delivery. J. Control. Release Off. J. Control. Release Soc..

[60] Barriga H.M.G., Pence I.J., Holme M.N., Doutch J.J., Penders J., Nele V., Thomas M.R., Carroni M., Stevens M.M. (2022). Coupling Lipid Nanoparticle Structure and Automated Single-Particle Composition Analysis to Design Phospholipase-Responsive Nanocarriers. Adv. Mater..

[61] Knudson C.J., Alves-Peixoto P., Muramatsu H., Stotesbury C., Tang L., Lin P.J.C., Tam Y.K., Weissman D., Pardi N., Sigal L.J. (2021). Lipid-nanoparticle-encapsulated mRNA vaccines induce protective memory CD8 T cells against a lethal viral infection. Mol. Ther. J. Am. Soc. Gene Ther..

[62] Basha G., Cottle A.G., Pretheeban T., Chan K.Y., Witzigmann D., Young R.N., Rossi F.M., Cullis P.R. (2022). Lipid nanoparticle-mediated silencing of osteogenic suppressor GNAS leads to osteogenic differentiation of mesenchymal stem cells in vivo. Mol. Ther. J. Am. Soc. Gene Ther..

[63] Shepherd S.J., Han X., Mukalel A.J., El-Mayta R., Thatte A.S., Wu J., Padilla M.S., Alameh M.G., Srikumar N., Lee D. (2023). Throughput-scalable manufacturing of SARS-CoV-2 mRNA lipid nanoparticle vaccines. Proc. Natl. Acad. Sci. USA.

[64] Xiang Y., Tian M., Huang J., Li Y., Li G., Li X., Jiang Z., Song X., Ma X. (2023). LMP2-mRNA lipid nanoparticle sensitizes EBV-related tumors to anti-PD-1 therapy by reversing T cell exhaustion. J. Nanobiotechnol..

[65] Spadea A., Jackman M., Cui L., Pereira S., Lawrence M.J., Campbell R.A., Ashford M. (2022). Nucleic Acid-Loaded Lipid Nanoparticle Interactions with Model Endosomal Membranes. ACS Appl. Mater. Interfaces.

[66] Zhang X., Jozic A., Song P., Xu Q., Shi X., Wang H., Bishop L., Struthers H.M., Rutledge J., Chen S. (2023). mRNA vaccine against fibroblast activation protein ameliorates murine models of inflammatory arthritis. Rheumatol. Immunol. Res..

[67] Pfeifle A., Thulasi Raman S.N., Lansdell C., Zhang W., Tamming L., Cecillon J., Laryea E., Patel D., Wu J., Gravel C. (2023). DNA lipid nanoparticle vaccine targeting outer surface protein C affords protection against homologous Borrelia burgdorferi needle challenge in mice. Front. Immunol..

[68] Ju Y., Lee W.S., Pilkington E.H., Kelly H.G., Li S., Selva K.J., Wragg K.M., Subbarao K., Nguyen T.H.O., Rowntree L.C. (2022). Anti-PEG Antibodies Boosted in Humans by SARS-CoV-2 Lipid Nanoparticle mRNA Vaccine. ACS Nano.

[69] Egan K.P., Hook L.M., Naughton A., Pardi N., Awasthi S., Cohen G.H., Weissman D., Friedman H.M. (2020). An HSV-2 nucleoside-modified mRNA genital herpes vaccine containing glycoproteins gC, gD, and gE protects mice against HSV-1 genital lesions and latent infection. PLoS Pathog..

[70] Nag K., Chandra Baray J., Rahman Khan M., Mahmud A., Islam J., Myti S., Ali R., Haq Sarker E., Kumar S., Hossain Chowdhury M. (2021). An mRNA-based vaccine candidate against SARS-CoV-2 elicits stable immuno-response with single dose. Vaccine.

[71] Kim D., Lai C.J., Cha I., Mahmud A., Islam J., Myti S., Ali R., Haq Sarker E., Kumar S., Hossain Chowdhury M. (2023). SFTSV Gn-Head mRNA vaccine confers efficient protection against lethal viral challenge. J. Med. Virol..

[72] Xia H., He Y.R., Zhan X.Y., Zha G.F. (2023). Mpox virus mRNA-lipid nanoparticle vaccine candidates evoke antibody responses and drive protection against the Vaccinia virus challenge in mice. Antivir. Res..

[73] Raffaele J., Loughney J.W., Rustandi R.R. (2022). Development of a microchip capillary electrophoresis method for determination of the purity and integrity of mRNA in lipid nanoparticle vaccines. Electrophoresis.

[74] Hori I., Harashima H., Yamada Y. (2023). Development of a Mitochondrial Targeting Lipid Nanoparticle Encapsulating Berberine. Int. J. Mol. Sci..

[75] Zamani P., Mashreghi M., Rezazade Bazaz M., Zargari S., Alizadeh F., Dorrigiv M., Abdoli A., Aminianfar H., Hatamipour M., Zarqi J. (2023). Characterization of stability, safety and immunogenicity of the mRNA lipid nanoparticle vaccine Iribovax against COVID-19 in nonhuman primates. J. Control Release.

[76] Nelson C.S., Jenks J.A., Pardi N., Goodwin M., Roark H., Edwards W., McLellan J.S., Pollara J., Weissman D., Permar S.R. (2020). Human Cytomegalovirus Glycoprotein B Nucleoside-Modified mRNA Vaccine Elicits Antibody Responses with Greater Durability and Breadth than MF59-Adjuvanted gB Protein Immunization. J. Virol..

[77] Lelis F., Byk L.A., Pustylnikov S., Nguyen V., Nguyen B., Nitz M., Tarte P., Tungare K., Li J., Manna S. (2023). Safety, immunogenicity and efficacy of an mRNA-based COVID-19 vaccine, GLB-COV2-043, in preclinical animal models. Sci. Rep..

[78] Narayanan E., Falcone S., Elbashir S.M., Attarwala H., Hassett K., Seaman M.S., Carfi A., Himansu S. (2022). Rational Design and In Vivo Characterization of mRNA-Encoded Broadly Neutralizing Antibody Combinations against HIV-1. Antibodies.

[79] Amici A., Pozzi D., Marchini C., Caracciolo G. (2023). The Transformative Potential of Lipid Nanoparticle-Protein Corona for Next-Generation Vaccines and Therapeutics. Mol. Pharm..

[80] Chivukula S., Plitnik T., Tibbitts T., Karve S., Dias A., Zhang D., Goldman R., Gopani H., Khanmohammed A., Sarode A. (2021). Development of multivalent mRNA vaccine candidates for seasonal or pandemic influenza. NPJ Vaccines.

[81] Melamed J.R., Hajj K.A., Chaudhary N., Strelkova D., Arral M.L., Pardi N., Alameh M.G., Miller J.B., Farbiak L., Siegwart D.J. (2022). Lipid nanoparticle chemistry determines how nucleoside base modifications alter mRNA delivery. J. Control. Release Off. J. Control. Release Soc..

[82] Chuang Y.M., Alameh M.G., Abouneameh S., Raduwan H., Ledizet M., Weissman D., Fikrig E. (2023). A mosquito AgTRIO mRNA vaccine contributes to immunity against malaria. NPJ Vaccines.

[83] Pardi N., LaBranche C.C., Ferrari G., Cain D.W., Tombácz I., Parks R.J., Muramatsu H., Mui B.L., Tam Y.K., Karikó K. (2019). Characterization of HIV-1 Nucleoside-Modified mRNA Vaccines in Rabbits and Rhesus Macaques. Mol. Ther. Nucleic Acids.

[84] Gouma S., Furey C., Santos J.J.S., Parkhouse K., Weirick M., Muramatsu H., Pardi N., Fan S.H.Y., Weissman D., Hensley S.E. (2023). Nucleoside-Modified mRNA-Based Influenza Vaccines Circumvent Problems Associated with H3N2 Vaccine Strain Egg Adaptation. J. Virol..

[85] John S., Yuzhakov O., Woods A., Deterling J., Hassett K., Shaw C.A., Ciaramella G. (2018). Multi-antigenic human cytomegalovirus mRNA vaccines that elicit potent humoral and cell-mediated immunity. Vaccine.

[86] Patra T., Meyer K., Haga Y., Reagan E.K., Weissman D., Ray R. (2023). Hepatitis C virus E1 and modified E2 delivered from an mRNA vaccine induces protective immunity. NPJ Vaccines.

[87] Zhao H., Wang T.C., Li X.F., Zhang N.N., Li L., Zhou C., Deng Y.Q., Cao T.S., Yang G., Li R.T. (2021). Long-term stability and protection efficacy of the RBD-targeting COVID-19 mRNA vaccine in nonhuman primates. Signal Transduct. Target. Ther..

[88] Medjmedj A., Ngalle-Loth A., Clemençon R., Hamacek J., Pichon C., Perche F. (2022). In Cellulo and In Vivo Comparison of Cholesterol, Beta-Sitosterol and Dioleylphosphatidylethanolamine for Lipid Nanoparticle Formulation of mRNA. Nanomaterials.

[89] El-Mayta R., Padilla M.S., Billingsley M.M. (2023). Testing the In Vitro and In Vivo Efficiency of mRNA-Lipid Nanoparticles Formulated by Microfluidic Mixing. J. Vis. Exp..

[90] Xu S., Zhang B., Yao J., Ruan W. (2023). A new H9 influenza virus mRNA vaccine elicits robust protective immunity against infection. Vaccine.

[91] Hook L.M., Awasthi S., Cairns T.M., Alameh M.G., Fowler B.T., Egan K.P., Sung M.M.H., Weissman D., Cohen G.H., Friedman H.M. (2022). Antibodies to Crucial Epitopes on HSV-2 Glycoprotein D as a Guide to Dosing an mRNA Genital Herpes Vaccine. Viruses.

[92] Ci L., Hard M., Zhang H., Gandham S., Hua S., Wickwire J., Wehrman T., Slauter R., Auerbach A., Kenney M. (2023). Biodistribution of Lipid 5, mRNA, and Its Translated Protein Following Intravenous Administration of mRNA-Encapsulated Lipid Nanoparticles in Rats. Drug Metab. Dispos..

[93] Maharjan R., Hada S., Lee J.E., Han H.K., Kim K.H., Seo H.J., Foged C., Jeong S.H. (2023). Comparative study of lipid nanoparticle-based mRNA vaccine bioprocess with machine learning and combinatorial artificial neural network-design of experiment approach. Int. J. Pharm..

[94] Ma Q., Li R., Guo J., Li M., Ma L., Dai J., Shi Y., Dai J., Huang Y., Dai C. (2022). Immunization with a Prefusion SARS-CoV-2 Spike Protein Vaccine (RBMRNA-176) Protects against Viral Challenge in Mice and Nonhuman Primates. Vaccines.

[95] Ma N., Xia Z.W., Zhang Z.G., Nian X.X., Li X.D., Gong Z., Zhang G.M., Le Y., Zhou R., Zhang J.Y. (2023). Development of an mRNA vaccine against a panel of heterologous H1N1 seasonal influenza viruses using a consensus hemagglutinin sequence. Emerg. Microbes Infect..

[96] Cui L., Hunter M.R., Sonzini S., Pereira S., Romanelli S.M., Liu K., Li W., Liang L., Yang B., Mahmoudi N. (2022). Mechanistic Studies of an Automated Lipid Nanoparticle Reveal Critical Pharmaceutical Properties Associated with Enhanced mRNA Functional Delivery In Vitro and In Vivo. Small.

[97] Wilhelmy C., Keil I.S., Uebbing L., Schroer M.A., Franke D., Nawroth T., Barz M., Sahin U., Haas H., Diken M. (2023). Polysarcosine-Functionalized mRNA Lipid Nanoparticles Tailored for Immunotherapy. Pharmaceutics.

[98] Dézsi L., Mészáros T., Kozma G., H-Velkei M., Oláh C.Z., Szabó M., Patkó Z., Fülöp T., Hennies M., Szebeni M. (2022). A naturally hypersensitive porcine model may help understand the mechanism of COVID-19 mRNA vaccine-induced rare (pseudo) allergic reactions: Complement activation as a possible contributing factor. Geroscience.

[99] Bai S., Yang T., Zhu C., Feng M., Zhang L., Zhang Z., Wang X., Yu R., Pan X., Zhao C. (2023). A single vaccination of nucleoside-modified Rabies mRNA vaccine induces prolonged highly protective immune responses in mice. Front. Immunol..

[100] Appelberg S., John L., Pardi N., Végvári Á., Bereczky S., Ahlén G., Monteil V., Abdurahman S., Mikaeloff F., Beattie M. (2022). Nucleoside-Modified mRNA Vaccines Protect IFNAR^−/−^ Mice against Crimean-Congo Hemorrhagic Fever Virus Infection. J. Virol..

[101] LaTourette P.C., Awasthi S., Desmond A., Pardi N., Cohen G.H., Weissman D., Friedman H.M. (2020). Protection against herpes simplex virus type 2 infection in a neonatal murine model using a trivalent nucleoside-modified mRNA in lipid nanoparticle vaccine. Vaccine.

[102] Ma Y., Fenton O.S. (2023). An Efficacy and Mechanism Driven Study on the Impact of Hypoxia on Lipid Nanoparticle Mediated mRNA Delivery. J. Am. Chem. Soc..

[103] Reinhart A.G., Osterwald A., Ringler P., Leiser Y., Lauer M.E., Martin R.E., Ullmer C., Schumacher F., Korn C., Keller M. (2023). Investigations into mRNA Lipid Nanoparticles Shelf-Life Stability under Nonfrozen Conditions. Mol. Pharm..

[104] Szebeni J., Storm G., Ljubimova J.Y., Castells M., Phillips E.J., Turjeman K., Barenholz Y., Crommelin D.J.A., Dobrovolskaia M.A. (2022). Applying lessons learned from nanomedicines to understand rare hypersensitivity reactions to mRNA-based SARS-CoV-2 vaccines. Nat. Nanotechnol..

[105] Hajiaghapour Asr M., Dayani F., Saedi Segherloo F., Kamedi A., Neill A.O., MacLoughlin R., Doroudian M. (2023). Lipid Nanoparticles as Promising Carriers for mRNA Vaccines for Viral Lung Infections. Pharmaceutics.

[106] Rohde C.M., Lindemann C., Giovanelli M., Sellers R.S., Diekmann J., Choudhary S., Ramaiah L., Vogel A.B., Chervona Y., Muik A. (2023). Toxicological Assessments of a Pandemic COVID-19 Vaccine-Demonstrating the Suitability of a Platform Approach for mRNA Vaccines. Vaccines.

[107] Zhu W., Wei L., Dong C., Wang Y., Kim J., Ma Y., Gonzalez G.X., Wang B.Z. (2022). cGAMP-adjuvanted multivalent influenza mRNA vaccines induce broadly protective immunity through cutaneous vaccination in mice. Mol. Ther. Nucleic Acids.

[108] Austin L.A., Smith J.S., Nahas D.D., Danzinger A., Secore S., O’Donnell G., Radcliffe S., Hu S., Perley J., Bett A.J. (2023). Split-Dose Administration Enhances Immune Responses Elicited by a mRNA/Lipid Nanoparticle Vaccine Expressing Respiratory Syncytial Virus F Protein. Mol. Pharm..

[109] Thaller A., Schmauder L., Frieß W., Winter G., Menzen T., Hawe A., Richter K., Winter G., Menzen T., Hawe A. (2023). SV-AUC as a stability-indicating method for the characterization of mRNA-LNPs. Eur. J. Pharm. Biopharm..

[110] Kim J., Jozic A., Lin Y., Eygeris Y., Bloom E., Tan X., Acosta C., MacDonald K.D., Welsher K.D., Sahay G. (2022). Engineering Lipid Nanoparticles for Enhanced Intracellular Delivery of mRNA through Inhalation. ACS Nano.

[111] Szebeni J., Kiss B., Bozó T., Turjeman K., Levi-Kalisman Y., Barenholz Y., Kellermayer M. (2023). Insights into the Structure of Comirnaty COVID-19 Vaccine: A Theory on Soft, Partially Bilayer-Covered Nanoparticles with Hydrogen Bond-Stabilized mRNA-Lipid Complexes. ACS Nano.

[112] Messerian K.O., Zverev A., Kramarczyk J.F., Zydney A.L. (2022). Pressure-dependent fouling behavior during sterile filtration of mRNA-containing lipid nanoparticles. Biotechnol. Bioeng..

[113] Wilson B., Geetha K.M. (2022). Lipid nanoparticles in the development of mRNA vaccines for COVID-19. J. Drug Deliv. Sci. Technol..

[114] Lazaros G., Klein A.L., Hatziantoniou S., Tsioufis C., Tsakris A., Anastassopoulou C. (2021). The Novel Platform of mRNA COVID-19 Vaccines and Myocarditis: Clues into the Potential Underlying Mechanism. Vaccine.

[115] Baharom F., Ramirez-Valdez R.A., Khalilnezhad A., Khalilnezhad S., Dillon M., Hermans D., Fussell S., Tobin K.K.S., Dutertre C.A., Lynn G.M. (2022). Systemic vaccination induces CD8+ T cells and remodels the tumor microenvironment. Cell.

[116] Carrasco M.J., Alishetty S., Alameh M.G., Said H., Wright L., Paige M., Soliman O., Weissman D., Cleveland T.E., Grishaev A. (2021). Ionization and structural properties of mRNA lipid nanoparticles influence expression in intramuscular and intravascular administration. Commun. Biol..

[117] Ly H.H., Daniel S., Soriano S.K.V., Kis Z., Blakney A.K. (2022). Optimization of Lipid Nanoparticles for saRNA Expression and Cellular Activation Using a Design-of-Experiment Approach. Mol. Pharm..

[118] Zhang H.L. (2023). Current status and patent prospective of lipid nanoparticle for mRNA delivery. Expert. Opin. Ther. Pat..

[119] Mu Z., Wiehe K., Saunders K.O., Henderson R., Cain D.W., Parks R., Martik D., Mansouri K., Edwards R.J., Newman A. (2022). mRNA-encoded HIV-1 Env trimer ferritin nanoparticles induce monoclonal antibodies that neutralize heterologous HIV-1 isolates in mice. Cell Rep..

[120] Li Z., Zhang X.Q., Ho W., Li F., Gao M., Bai X., Xu X. (2022). Enzyme-Catalyzed One-Step Synthesis of Ionizable Cationic Lipids for Lipid Nanoparticle-Based mRNA COVID-19 Vaccines. ACS Nano.

[121] Liu G.W., Guzman E.B., Menon N., Langer R.S. (2023). Lipid Nanoparticles for Nucleic Acid Delivery to Endothelial Cells. Pharm. Res..

[122] Willis E., Pardi N., Parkhouse K., Mui B.L., Tam Y.K., Weissman D., Hensley S.E. (2020). Nucleoside-modified mRNA vaccination partially overcomes maternal antibody inhibition of de novo immune responses in mice. Sci. Transl. Med..

[123] Melzi E., Willis J.R., Ma K.M., Lin Y.C., Kratochvil S., Berndsen Z.T., Landais E.A., Kalyuzhniy O., Nair U., Warner J. (2022). Membrane-bound mRNA immunogens lower the threshold to activate HIV Env V2 apex-directed broadly neutralizing B cell precursors in humanized mice. Immunity.

[124] Ma Y., Fenton O.S. (2023). A Unified Strategy to Improve Lipid Nanoparticle Mediated mRNA Delivery Using Adenosine Triphosphate. J. Am. Chem. Soc..

[125] Everton E., Rizvi F., Smith A.R., Beattie M., Tam Y., Pardi N., Weissman D., Gouon-Evans V. (2021). Transient yet Robust Expression of Proteins in the Mouse Liver via Intravenous Injection of Lipid Nanoparticle-encapsulated Nucleoside-modified mRNA. Bio-Protocol.

[126] Lindgren G., Ols S., Liang F., Thompson E.A., Lin A., Hellgren F., Bahl K., John S., Yuzhakov O., Hassett K.J. (2017). Induction of Robust B Cell Responses after Influenza mRNA Vaccination Is Accompanied by Circulating Hemagglutinin-Specific ICOS+ PD-1+ CXCR3+ T Follicular Helper Cells. Front. Immunol..

[127] Shirane D., Tanaka H., Sakurai Y., Taneichi S., Nakai Y., Tange K., Ishii I., Akita H. (2023). Development of an Alcohol Dilution-Lyophilization Method for the Preparation of mRNA-LNPs with Improved Storage Stability. Pharmaceutics.

[128] Vergani E., Daveri E., Vallacchi V., Bergamaschi L., Lalli L., Castelli C., Rodolfo M., Rivoltini L., Huber V. (2022). Extracellular vesicles in anti-tumor immunity. Semin. Cancer Biol..

[129] Hou X., Zaks T., Langer R., Dong Y. (2021). Lipid nanoparticles for mRNA delivery. Nat. Rev. Mater..

[130] Wang C., Zhang Y., Dong Y. (2021). Lipid Nanoparticle-mRNA Formulations for Therapeutic Applications. Acc. Chem. Res..

[131] Xu L., Wang X., Wang W., Sun M., Choi W.J., Kim J.Y., Hao C., Li S., Qu A., Lu M. (2022). Enantiomer-dependent immunological response to chiral nanoparticles. Nature.

[132] Ogawa K., Kato N., Yoshida M., Hiu T., Matsuo T., Mizukami S., Omata D., Suzuki R., Maruyama K., Mukai H. (2022). Focused ultrasound/microbubbles-assisted BBB opening enhances LNP-mediated mRNA delivery to brain. J. Control Release.

[133] Khare P., Edgecomb S.X., Hamadani C.M., Tanner E.E.L., Manickam D.S. (2023). Lipid nanoparticle-mediated drug delivery to the brain. Adv. Drug Deliv. Rev..

[134] Jhunjhunwala S., Hammer C., Delamarre L. (2021). Antigen presentation in cancer: Insights into tumour immunogenicity and immune evasion. Nat. Rev. Cancer.

[135] Kazemian P., Yu S.Y., Thomson S.B., Birkenshaw A., Leavitt B.R., Ross C.J.D. (2022). Lipid-Nanoparticle-Based Delivery of CRISPR/Cas9 Genome-Editing Components. Mol. Pharm..

[136] Friis K.P., Gracin S., Oag S., Leijon A., Sand E., Lindberg B., Lázaro-Ibáñez E., Lindqvist J., Whitehead K.A., Bak A. (2023). Spray dried lipid nanoparticle formulations enable intratracheal delivery of mRNA. J. Control. Release Off. J. Control. Release Soc..

[137] Dobrowolski C., Paunovska K., Schrader Echeverri E., Loughrey D., Da Silva Sanchez A.J., Ni H., Hatit M.Z.C., Lokugamage M.P., Kuzminich Y., Peck H.E. (2022). Nanoparticle single-cell multiomic readouts reveal that cell heterogeneity influences lipid nanoparticle-mediated messenger RNA delivery. Nat. Nanotechnol..

[138] Al Subeh Z.Y., Poschel D.B., Redd P.S., Klement J.D., Merting A.D., Yang D., Mehta M., Shi H., Colson Y.L., Oberlies N.H. (2022). Lipid Nanoparticle Delivery of Fas Plasmid Restores Fas Expression to Suppress Melanoma Growth In Vivo. ACS Nano.

[139] Nakamura T., Sato Y., Yamada Y., Abd Elwakil M.M., Kimura S., Younis M.A., Harashima H. (2022). Extrahepatic targeting of lipid nanoparticles in vivo with intracellular targeting for future nanomedicines. Adv. Drug Deliv. Rev..

[140] Anderluzzi G., Lou G., Woods S., Schmidt S.T., Gallorini S., Brazzoli M., Johnson R., Roberts C.W., O’Hagan D.T., Baudner B.C. (2022). The role of nanoparticle format and route of administration on self-amplifying mRNA vaccine potency. J. Control. Release Off. J. Control. Release Soc..

[141] Yi Y., Yu M., Li W., Zhu D., Mei L., Ou M. (2023). Vaccine-like nanomedicine for cancer immunotherapy. J. Control. Release Off. J. Control. Release Soc..

[142] Huang T., Peng L., Han Y., Wang D., He X., Wang J., Ou C. (2022). Lipid nanoparticle-based mRNA vaccines in cancers: Current advances and future prospects. Front. Immunol..

[143] Tan J.Y.B., Yoon B.K., Cho N.J., Lovrić J., Jug M., Jackman J.A. (2021). Lipid Nanoparticle Technology for Delivering Biologically Active Fatty Acids and Monoglycerides. Int. J. Mol. Sci..

[144] Daly O., Mahiny A.J., Majeski S., McClintock K., Reichert J., Boros G., Szabó G.T., Reinholz J., Schreiner P., Reid S. (2023). ASL mRNA-LNP Therapeutic for the Treatment of Argininosuccinic Aciduria Enables Survival Benefit in a Mouse Model. Biomedicines.

[145] Verma A.K., Perlman S. (2022). Lipid nanoparticle-mRNA: Another step in the fight against COVID-19. Cell Res..

[146] Yin T., Xin H., Yu J., Teng F. (2021). The role of exosomes in tumour immunity under radiotherapy: Eliciting abscopal effects?. Biomark. Res..

[147] Massaro M., Wu S., Baudo G., Liu H., Collum S., Lee H., Stigliano C., Segura-Ibarra V., Karmouty-Quintana H., Blanco E. (2023). Lipid nanoparticle-mediated mRNA delivery in lung fibrosis. Eur. J. Pharm. Sci. Off. J. Eur. Fed. Pharm. Sci..

[148] Xu X., Wang X., Liao Y.P., Luo L., Xia T., Nel A.E. (2023). Use of a Liver-Targeting Immune-Tolerogenic mRNA Lipid Nanoparticle Platform to Treat Peanut-Induced Anaphylaxis by Single and Multiple-Epitope Nucleotide Sequence Delivery. ACS Nano.

[149] Tam A., Kulkarni J., An K., Li L., Dorscheid D.R., Singhera G.K., Bernatchez P., Reid G., Chan K., Witzigmann D. (2022). Lipid nanoparticle formulations for optimal RNA-based topical delivery to murine airways. Eur. J. Pharm. Sci. Off. J. Eur. Fed. Pharm. Sci..

[150] Nakamura T., Sato T., Endo R., Sasaki S., Takahashi N., Sato Y., Hyodo M., Hayakawa Y., Harashima H. (2021). STING agonist loaded lipid nanoparticles overcome anti-PD-1 resistance in melanoma lung metastasis via NK cell activation. J. Immunother. Cancer.

[151] Zhang Y., Hou X., Du S., Xue Y., Yan J., Kang D.D., Zhong Y., Wang C., Deng B., McComb D.W. (2023). Close the cancer-immunity cycle by integrating lipid nanoparticle-mRNA formulations and dendritic cell therapy. Nat. Nanotechnol..

[152] Tanaka H., Hagiwara S., Shirane D., Yamakawa T., Sato Y., Matsumoto C., Ishizaki K., Hishinuma M., Chida K., Sasaki K. (2023). Ready-to-Use-Type Lyophilized Lipid Nanoparticle Formulation for the Postencapsulation of Messenger RNA. ACS Nano.

[153] Blakney A.K., McKay P.F., Hu K., Samnuan K., Jain N., Brown A., Thomas A., Rogers P., Polra K., Sallah H. (2021). Polymeric and lipid nanoparticles for delivery of self-amplifying RNA vaccines. J. Control. Release Off. J. Control. Release Soc..

[154] Ju H., Kim D., Oh Y.K. (2022). Lipid nanoparticle-mediated CRISPR/Cas9 gene editing and metabolic engineering for anticancer immunotherapy. Asian J. Pharm. Sci..

[155] Meulewaeter S., Nuytten G., Cheng M.H.Y., De Smedt S.C., Cullis P.R., De Beer T., Lentacker I., Verbeke R. (2023). Continuous freeze-drying of messenger RNA lipid nanoparticles enables storage at higher temperatures. J. Control. Release Off. J. Control. Release Soc..

[156] Golubovskaya V., Sienkiewicz J., Sun J., Huang Y., Hu L., Zhou H., Harto H., Xu S., Berahovich R., Bodmer W. (2023). mRNA-Lipid Nanoparticle (LNP) Delivery of Humanized EpCAM-CD3 Bispecific Antibody Significantly Blocks Colorectal Cancer Tumor Growth. Cancers.

[157] Zhou J.E., Sun L., Jia Y., Wang Z., Luo T., Tan J., Fang X., Zhu H., Wang J., Yu L. (2022). Lipid nanoparticles produce chimeric antigen receptor T cells with interleukin-6 knockdown in vivo. J. Control. Release Off. J. Control. Release Soc..

[158] Herrera-Barrera M., Gautam M., Lokras A., Vlasova K., Foged C., Sahay G. (2023). Lipid Nanoparticle-Enabled Intracellular Delivery of Prime Editors. AAPS J..

[159] Brader M.L., Williams S.J., Banks J.M., Hui W.H., Zhou Z.H., Jin L. (2021). Encapsulation state of messenger RNA inside lipid nanoparticles. Biophys. J..

[160] Lam K., Leung A., Martin A., Wood M., Schreiner P., Palmer L., Daly O., Zhao W., McClintock K., Heyes J. (2023). Unsaturated, Trialkyl Ionizable Lipids are Versatile Lipid-Nanoparticle Components for Therapeutic and Vaccine Applications. Adv. Mater..

[161] Driscoll D.F. (2022). Lipid nanoparticle-based COVID-19 vaccines: Concerns about stability. Am. J. Health-Syst. Pharm. AJHP Off. J. Am. Soc. Health-Syst. Pharm..

[162] Álvarez-Benedicto E., Farbiak L., Márquez Ramírez M., Wang X., Johnson L.T., Mian O., Guerrero E.D., Siegwart D.J. (2023). Optimization of phospholipid chemistry for improved lipid nanoparticle (LNP) delivery of messenger RNA (mRNA). Biomater. Sci..

[163] Huayamares S.G., Lokugamage M.P., Rab R., Da Silva Sanchez A.J., Kim H., Radmand A., Loughrey D., Lian L., Hou Y., Achyut B.R. (2023). High-throughput screens identify a lipid nanoparticle that preferentially delivers mRNA to human tumors in vivo. J. Control. Release Off. J. Control. Release Soc..

[164] Kheirolomoom A., Kare A.J., Ingham E.S., Paulmurugan R., Robinson E.R., Baikoghli M., Inayathullah M., Seo J.W., Wang J., Fite B.Z. (2022). In situ T-cell transfection by anti-CD3-conjugated lipid nanoparticles leads to T-cell activation, migration, and phenotypic shift. Biomaterials.

[165] Chen W., Chen Y., Ren Y., Gao C., Ning C., Deng H., Li P., Ma Y., Li H., Fu L. (2022). Lipid nanoparticle-assisted miR29a delivery based on core-shell nanofibers improves tendon healing by cross-regulation of the immune response and matrix remodeling. Biomaterials.

[166] Chen Y., Chen W., Ren Y., Li S., Liu M., Xing J., Han Y., Chen Y., Tao R., Guo L. (2022). Lipid nanoparticle-encapsulated VEGFa siRNA facilitates cartilage formation by suppressing angiogenesis. Int. J. Biol. Macromol..

[167] Fan N., Chen K., Zhu R., Zhang Z., Huang H., Qin S., Zheng Q., He Z., He X., Xiao W. (2022). Manganese-coordinated mRNA vaccines with enhanced mRNA expression and immunogenicity induce robust immune responses against SARS-CoV-2 variants. Sci. Adv..

[168] LoPresti S.T., Arral M.L., Chaudhary N., Whitehead K.A. (2022). The replacement of helper lipids with charged alternatives in lipid nanoparticles facilitates targeted mRNA delivery to the spleen and lungs. J. Control. Release Off. J. Control. Release Soc..

[169] Pine M., Arora G., Hart T.M., Bettini E., Gaudette B.T., Muramatsu H., Tombácz I., Kambayashi T., Tam Y.K., Brisson D. (2023). Development of an mRNA-lipid nanoparticle vaccine against Lyme disease. Mol. Ther. J. Am. Soc. Gene Ther..

[170] Arevalo C.P., Bolton M.J., Le Sage V., Ye N., Furey C., Muramatsu H., Alameh M.G., Pardi N., Drapeau E.M., Parkhouse K. (2022). A multivalent nucleoside-modified mRNA vaccine against all known influenza virus subtypes. Science.

[171] Tenchov R., Sasso J.M., Zhou Q.A. (2023). PEGylated Lipid Nanoparticle Formulations: Immunological Safety and Efficiency Perspective. Bioconjug. Chem..

[172] Vlatkovic I. (2021). Non-Immunotherapy Application of LNP-mRNA: Maximizing Efficacy and Safety. Biomedicines.

[173] Maugeri M., Nawaz M., Papadimitriou A., Angerfors A., Camponeschi A., Na M., Hölttä M., Skantze P., Johansson S., Sundqvist M. (2019). Linkage between endosomal escape of LNP-mRNA and loading into EVs for transport to other cells. Nat. Commun..

[174] Kenjo E., Hozumi H., Makita Y., Iwabuchi K.A., Fujimoto N., Matsumoto S., Kimura M., Amano Y., Ifuku M., Naoe Y. (2021). Low immunogenicity of LNP allows repeated administrations of CRISPR-Cas9 mRNA into skeletal muscle in mice. Nat. Commun..

[175] Li Y., Ma X., Yue Y., Zhang K., Cheng K., Feng Q., Ma N., Liang J., Zhang T., Zhang L. (2022). Rapid Surface Display of mRNA Antigens by Bacteria-Derived Outer Membrane Vesicles for a Personalized Tumor Vaccine. Adv. Mater..

[176] Peng L., Fang Z., Renauer P.A., McNamara A., Park J.J., Lin Q., Zhou X., Dong M.B., Zhu B., Zhao H. (2022). Multiplexed LNP-mRNA vaccination against pathogenic coronavirus species. Cell Rep..

[177] Tsiambas E., Chrysovergis A., Papanikolaou V., Mastronikolis N., Ragos V., Batistatou A., Peschos D., Kavantzas N., Lazaris A.C., Kyrodimos E. (2021). Impact of Ribosome Activity on SARS-CoV-2 LNP—Based mRNA Vaccines. Front. Mol. Biosci..

[178] Bahl K., Senn J.J., Yuzhakov O., Bulychev A., Brito L.A., Hassett K.J., Laska M.E., Smith M., Almarsson Ö., Thompson J. (2017). Preclinical and Clinical Demonstration of Immunogenicity by mRNA Vaccines against H10N8 and H7N9 Influenza Viruses. Mol. Ther..

[179] Patel S.K., Billingsley M.M., Frazee C., Han X., Swingle K.L., Qin J., Alameh M.G., Wang K., Weissman D., Mitchell M.J. (2022). Hydroxycholesterol substitution in ionizable lipid nanoparticles for mRNA delivery to T cells. J. Control Release.

[180] Gao K., Li J., Song H., Han H., Wang Y., Yin B., Farmer D.L., Murthy N., Wang A. (2023). In utero delivery of mRNA to the heart, diaphragm and muscle with lipid nanoparticles. Bioact. Mater..

[181] Ball R.L., Hajj K.A., Vizelman J., Bajaj P., Whitehead K.A. (2018). Lipid Nanoparticle Formulations for Enhanced Co-delivery of siRNA and mRNA. Nano Lett..

[182] Bogaert B., Sauvage F., Guagliardo R., Muntean C., Nguyen V.P., Pottie E., Wels M., Minnaert A.K., De Rycke R., Yang Q. (2022). A lipid nanoparticle platform for mRNA delivery through repurposing of cationic amphiphilic drugs. J. Control Release.

[183] Papi M., Pozzi D., Palmieri V., Caracciolo G. (2022). Principles for optimization and validation of mRNA lipid nanoparticle vaccines against COVID-19 using 3D bioprinting. Nano Today..

[184] Da Silva Sanchez A.J., Zhao K., Huayamares S.G., Hatit M.Z.C., Lokugamage M.P., Loughrey D., Dobrowolski C., Wang S., Kim H., Paunovska K. (2023). Substituting racemic ionizable lipids with stereopure ionizable lipids can increase mRNA delivery. J. Control Release.

[185] Swingle K.L., Safford H.C., Geisler H.C., Hamilton A.G., Thatte A.S., Billingsley M.M., Joseph R.A., Mrksich K., Padilla M.S., Ghalsasi A.A. (2023). Ionizable Lipid Nanoparticles for In Vivo mRNA Delivery to the Placenta during Pregnancy. J. Am. Chem. Soc..

[186] Attarwala H., Lumley M., Liang M., Ivaturi V., Senn J. (2023). Translational Pharmacokinetic/Pharmacodynamic Model for mRNA-3927, an Investigational Therapeutic for the Treatment of Propionic Acidemia. Nucleic Acid. Ther..

[187] Miao L., Lin J., Huang Y., Li L., Delcassian D., Ge Y., Shi Y., Anderson D.G. (2020). Synergistic lipid compositions for albumin receptor mediated delivery of mRNA to the liver. Nat. Commun..

[188] Liang F., Lindgren G., Lin A., Thompson E.A., Ols S., Röhss J., John S., Hassett K., Yuzhakov O., Bahl K. (2017). Efficient Targeting and Activation of Antigen-Presenting Cells In Vivo after Modified mRNA Vaccine Administration in Rhesus Macaques. Mol. Ther..

[189] Ryals R.C., Patel S., Acosta C., McKinney M., Pennesi M.E., Sahay G. (2020). The effects of PEGylation on LNP based mRNA delivery to the eye. PLoS ONE.

[190] Zhuang X., Qi Y., Wang M., Yu N., Nan F., Zhang H., Tian M., Li C., Lu H., Jin N. (2020). mRNA Vaccines Encoding the HA Protein of Influenza A H1N1 Virus Delivered by Cationic Lipid Nanoparticles Induce Protective Immune Responses in Mice. Vaccines.

[191] Sinegra A.J., Evangelopoulos M., Park J., Huang Z., Mirkin C.A. (2021). Lipid Nanoparticle Spherical Nucleic Acids for Intracellular DNA and RNA Delivery. Nano Lett..

[192] Zhang Y., Yan J., Hou X., Wang C., Kang D.D., Xue Y., Du S., Deng B., McComb D.W., Liu S.L. (2023). STING Agonist-Derived LNP-mRNA Vaccine Enhances Protective Immunity Against SARS-CoV-2. Nano Lett..

[193] Long J., Wang D., Wang A., Chen P., Lin Y., Bian J., Yang X., Zheng M., Zhang H., Zheng Y. (2022). A mutation-based gene set predicts survival benefit after immunotherapy across multiple cancers and reveals the immune response landscape. Genome Med..

[194] Lckenstein L.M., Garidel P. (2019). Lipid-based nanoparticle formulations for small molecules and RNA drugs. Expert. Opin. Drug Deliv..

[195] Semple S.C., Leone R., Barbosa C.J., Tam Y.K., Lin P.J.C. (2022). Lipid Nanoparticle Delivery Systems to Enable mRNA-Based Therapeutics. Pharmaceutics.

[196] Cui L., Pereira S., Sonzini S., Van Pelt S., Romanelli S.M., Liang L., Ulkoski D., Krishnamurthy V.R., Brannigan E., Brankin C. (2022). Development of a high-throughput platform for screening lipid nanoparticles for mRNA delivery. Nanoscale.

[197] Swetha K., Kotla N.G., Tunki L., Jayaraj A., Bhargava S.K., Hu H., Bonam S.R., Kurapati R. (2023). Recent Advances in the Lipid Nanoparticle-Mediated Delivery of mRNA Vaccines. Vaccines.

[198] Wang M., Huang Y., Chen M., Wang W., Wu F., Zhong T., Chen X., Wang F., Li Y., Yu J. (2023). Inhibition of tumor intrinsic BANF1 activates antitumor immune responses via cGAS-STING and enhances the efficacy of PD-1 blockade. J. Immunother. Cancer.

[199] Zhuang X., Chen L., Yang S., Xia S., Xu Z., Zhang T., Zeng B., Yu T., Yu N., Wang W. (2022). R848 Adjuvant Laden With Self-Assembled Nanoparticle-Based mRNA Vaccine Elicits Protective Immunity Against H5N1 in Mice. Front. Immunol..

[200] Fang Z., Peng L., Filler R., Suzuki K., McNamara A., Lin Q., Renauer P.A., Yang L., Menasche B., Sanchez A. (2022). Omicron-specific mRNA vaccination alone and as a heterologous booster against SARS-CoV-2. Nat. Commun..

[201] Radloff K., Gutbier B., Dunne C.M., Moradian H., Schwestka M., Gossen M., Ahrens K., Kneller L., Wang Y., Moga A. (2023). Cationic LNP-formulated mRNA expressing Tie2-agonist in the lung endothelium prevents pulmonary vascular leakage. Mol. Ther. Nucleic Acids.

[202] August A., Brito L., Paris R., Zaks T. (2022). Clinical Development of mRNA Vaccines: Challenges and Opportunities. Curr. Top. Microbiol. Immunol..

[203] Fedorowski J.J. (2021). Could amantadine interfere with COVID-19 vaccines based on the LNP-mRNA platform?. Arch. Med. Sci..

[204] Somiya M., Mine S., Yasukawa K., Ikeda S. (2021). Sex differences in the incidence of anaphylaxis to LNP-mRNA COVID-19 vaccines. Vaccine.

[205] Wang W., Feng S., Ye Z., Gao H., Lin J., Ouyang D. (2022). Prediction of lipid nanoparticles for mRNA vaccines by the machine learning algorithm. Acta Pharm. Sin. B.

[206] Leung J., Strong C., Badior K.E., Robertson M., Wu X., Meledeo M.A., Kang E., Paul M., Sato Y., Harashima H. (2023). Genetically engineered transfusable platelets using mRNA lipid nanoparticles. Sci. Adv..

[207] Novakowski S., Jiang K., Prakash G., Kastrup C. (2019). Delivery of mRNA to platelets using lipid nanoparticles. Sci. Rep..

[208] Sayers E.J., Peel S.E., Schantz A., England R.M., Beano M., Bates S.M., Desai A.S., Puri S., Ashford M.B., Jones A.T. (2019). Endocytic Profiling of Cancer Cell Models Reveals Critical Factors Influencing LNP-Mediated mRNA Delivery and Protein Expression. Mol. Ther..

[209] Wu L., Wang W., Tian J., Qi C., Cai Z., Yan W., Xuan S., Shang A. (2021). Engineered mRNA-expressed bispecific antibody prevent intestinal cancer via lipid nanoparticle delivery. Bioengineered.

[210] Zeng Y., Escalona-Rayo O., Knol R., Kros A., Slütter B. (2023). Lipid nanoparticle-based mRNA candidates elicit potent T cell responses. Biomater. Sci..

[211] Wang Y., Si X., Feng Y., Feng D., Xu X., Zhang Y. (2023). Ionizable Lipids with Triazole Moiety from Click Reaction for LNP-Based mRNA Delivery. Molecules.

[212] Ramos da Silva J., Bitencourt Rodrigues K., Formoso Pelegrin G., Silva Sales N., Muramatsu H., de Oliveira Silva M., Porchia B.F.M.M., Moreno A.C.R., Aps L.R.M.M., Venceslau-Carvalho A.A. (2023). Single immunizations of self-amplifying or non-replicating mRNA-LNP vaccines control HPV-associated tumors in mice. Sci. Transl. Med..

[213] Provine N.M., Klenerman P. (2023). Adenovirus vector and mRNA vaccines: Mechanisms regulating their immunogenicity. Eur. J. Immunol..

[214] Qin J., Xue L., Gong N., Zhang H., Shepherd S.J., Haley R.M., Swingle K.L., Mitchell M.J. (2022). RGD peptide-based lipids for targeted mRNA delivery and gene editing applications. RSC Adv..

[215] Goswami R., Chatzikleanthous D., Lou G., Giusti F., Bonci A., Taccone M., Brazzoli M., Gallorini S., Ferlenghi I., Berti F. (2019). Mannosylation of LNP Results in Improved Potency for Self-Amplifying RNA (SAM) Vaccines. ACS Infect. Dis..

[216] Vigil T.N., Zhang-Hulsey D., Santos J.L., Patrick Hussmann G. (2021). Expediting in vitro characterization of mRNA-based gene therapies via high-content fluorescent imaging. Anal. Biochem..

[217] Zhang Y., Wang J., Xing H., Liu C., Zha W., Dong S., Jiang Y., Li X. (2023). Enhanced immunogenicity induced by mRNA vaccines with various lipid nanoparticles as carriers for SARS-CoV-2 infection. J. Mater. Chem. B.

[218] Freyn A.W., Ramos da Silva J., Rosado V.C., Bliss C.M., Pine M., Mui B.L., Tam Y.K., Madden T.D., de Souza Ferreira L.C., Weissman D. (2020). A Multi-Targeting, Nucleoside-Modified mRNA Influenza Virus Vaccine Provides Broad Protection in Mice. Mol. Ther. J. Am. Soc. Gene Ther..

[219] Huo H., Cheng X., Xu J., Lin J., Chen N., Lu X. (2023). A fluorinated ionizable lipid improves the mRNA delivery efficiency of lipid nanoparticles. J. Mater. Chem. B.

[220] Diaz-Trelles R., Perez-Garcia C.G. (2022). Present and future of lipid nanoparticle-mRNA technology in phenylketonuria disease treatment. Int. Rev. Cell Mol. Biol..

[221] Long J., Yu C., Zhang H., Cao Y., Sang Y., Lu H., Zhang Z., Wang X., Wang H., Song G. (2023). Novel Ionizable Lipid Nanoparticles for SARS-CoV-2 Omicron mRNA Delivery. Adv. Healthc. Mater..

[222] Chang D.F., Court K.A., Holgate R., Davis E.A., Bush K.A., Quick A.P., Spiegel A.J., Rahimi M., Cooke J.P., Godin B. (2023). Telomerase mRNA Enhances Human Skin Engraftment for Wound Healing. Adv. Healthc. Mater..

[223] VanBlargan L.A., Himansu S., Foreman B.M., Ebel G.D., Pierson T.C., Diamond M.S. (2018). An mRNA Vaccine Protects Mice against Multiple Tick-Transmitted Flavivirus Infections. Cell Rep..

[224] Żak M.M., Kaur K., Yoo J., Kurian A.A., Adjmi M., Mainkar G., Yoon S., Zangi L. (2023). Modified mRNA Formulation and Stability for Cardiac and Skeletal Muscle Delivery. Pharmaceutics.

[225] Cao W., Xia T. (2023). mRNA lipid nanoparticles induce immune tolerance to treat human diseases. Med. Rev..

[226] Bähr-Mahmud H., Ellinghaus U., Stadler C.R., Fischer L., Lindemann C., Chaturvedi A., Diekmann J., Wöll S., Biermann I., Hebich B. (2023). Preclinical characterization of an mRNA-encoded anti-Claudin 18.2 antibody. Oncoimmunology.

[227] Swingle K.L., Billingsley M.M., Bose S.K., White B., Palanki R., Dave A., Patel S.K., Gong N., Hamilton A.G., Alameh M.G. (2022). Amniotic fluid stabilized lipid nanoparticles for in utero intra-amniotic mRNA delivery. J. Control Release.

[228] Yihunie W., Nibret G., Aschale Y. (2023). Recent Advances in Messenger Ribonucleic Acid (mRNA) Vaccines and Their Delivery Systems: A Review. Clin. Pharmacol..

[229] Szőke D., Kovács G., Kemecsei É., Bálint L., Szoták-Ajtay K., Aradi P., Styevkóné Dinnyés A., Mui B.L., Tam Y.K., Madden T.D. (2021). Nucleoside-modified VEGFC mRNA induces organ-specific lymphatic growth and reverses experimental lymphedema. Nat. Commun..

[230] Pardi N., Weissman D. (2017). Nucleoside Modified mRNA Vaccines for Infectious Diseases. Methods Mol. Biol..

[231] Sang Y., Zhang Z., Liu F., Lu H., Yu C., Sun H., Long J., Cao Y., Mai J., Miao Y. (2023). Monkeypox virus quadrivalent mRNA vaccine induces immune response and protects against vaccinia virus. Signal Transduct. Target. Ther..

[232] Broudic K., Amberg A., Schaefer M., Spirkl H.P., Bernard M.C., Desert P. (2022). Nonclinical safety evaluation of a novel ionizable lipid for mRNA delivery. Toxicol. Appl. Pharmacol..

[233] Fekete S., Doneanu C., Addepalli B., Gaye M., Nguyen J., Alden B., Birdsall R., Han D., Isaac G., Lauber M. (2023). Challenges and emerging trends in liquid chromatography-based analyses of mRNA pharmaceuticals. J. Pharm. Biomed. Anal..

[234] Sun M., Dang U.J., Yuan Y., Psaras A.M., Osipitan O., Brooks T.A., Lu F., Di Pasqua A.J. (2022). Optimization of DOTAP/chol Cationic Lipid Nanoparticles for mRNA, pDNA, and Oligonucleotide Delivery. AAPS PharmSciTech..

[235] McCrudden C.M., Bennie L., Chambers P., Wilson J., Kerr M., Ziminska M., Douglas H., Kuhn S., Carroll E., O’Brien G. (2023). Peptide delivery of a multivalent mRNA SARS-CoV-2 vaccine. J. Control Release.

[236] Thran M., Mukherjee J., Pönisch M., Fiedler K., Thess A., Mui B.L., Hope M.J., Tam Y.K., Horscroft N., Heidenreich R. (2017). mRNA mediates passive vaccination against infectious agents, toxins, and tumors. EMBO Mol. Med..

[237] Zhang J., Shrivastava S., Cleveland R.O., Rabbitts T.H. (2019). Lipid-mRNA Nanoparticle Designed to Enhance Intracellular Delivery Mediated by Shock Waves. ACS Appl. Mater. Interfaces.

[238] Nakamura T., Nakade T., Sato Y., Harashima H. (2023). Delivering mRNA to a human NK cell line, NK-92 cells, by lipid nanoparticles. Int. J. Pharm..

[239] Huysmans H., Zhong Z., De Temmerman J., Mui B.L., Tam Y.K., Mc Cafferty S., Gitsels A., Vanrompay D., Sanders N.N. (2019). Expression Kinetics and Innate Immune Response after Electroporation and LNP-Mediated Delivery of a Self-Amplifying mRNA in the Skin. Mol. Ther. Nucleic Acids.

[240] Dong S., Wang J., Guo Z., Zhang Y., Wang Y., Dong S., Liu C., Xing H., Li X. (2023). Efficient delivery of VEGFA mRNA for promoting wound healing via ionizable lipid nanoparticles. Bioorg Med. Chem..

[241] Zhao Z., He S., Yu X., Lai X., Tang S., Mariya M E.A., Wang M., Yan H., Huang X., Zeng S. (2022). Analysis and Experimental Validation of Rheumatoid Arthritis Innate Immunity Gene CYFIP2 and Pan-Cancer. Front. Immunol..

[242] Yamazaki K., Kubara K., Ishii S., Kondo K., Suzuki Y., Miyazaki T., Mitsuhashi K., Ito M., Tsukahara K. (2023). Lipid nanoparticle-targeted mRNA formulation as a treatment for ornithine-transcarbamylase deficiency model mice. Mol. Ther. Nucleic Acids.

[243] Patel S., Ashwanikumar N., Robinson E., DuRoss A., Sun C., Murphy-Benenato K.E., Mihai C., Almarsson Ö., Sahay G. (2017). Boosting Intracellular Delivery of Lipid Nanoparticle-Encapsulated mRNA. Nano Lett..

[244] Olson K.E., Namminga K.L., Lu Y., Thurston M.J., Schwab A.D., De Picciotto S., Tse S.W., Walker W., Iacovelli J., Small C. (2021). Granulocyte-macrophage colony-stimulating factor mRNA and Neuroprotective Immunity in Parkinson’s disease. Biomaterials.

[245] Nawaz M., Heydarkhan-Hagvall S., Tangruksa B., González-King Garibotti H., Jing Y., Maugeri M., Kohl F., Hultin L., Reyahi A., Camponeschi A. (2023). Lipid Nanoparticles Deliver the Therapeutic VEGFA mRNA In Vitro and In Vivo and Transform Extracellular Vesicles for Their Functional Extensions. Adv. Sci..

[246] Popowski K.D., López de Juan Abad B., George A., Silkstone D., Belcher E., Chung J., Ghodsi A., Lutz H., Davenport J., Flanagan M. (2022). Inhalable exosomes outperform liposomes as mRNA and protein drug carriers to the lung. Extracell. Vesicle.

[247] Safford H.C., Swingle K.L., Geisler H.C., Hamilton A.G., Thatte A.S., Ghalsasi A.A., Billingsley M.M., Alameh M.G., Weissman D., Mitchell M.J. (2023). Orthogonal Design of Experiments for Engineering of Lipid Nanoparticles for mRNA Delivery to the Placenta. Small.

[248] Lokugamage M.P., Gan Z., Zurla C., Levin J., Islam F.Z., Kalathoor S., Sato M., Sago C.D., Santangelo P.J., Dahlman J.E. (2020). Mild Innate Immune Activation Overrides Efficient Nanoparticle-Mediated RNA Delivery. Adv. Mater..

[249] Zhdanov V.P. (2017). Kinetics of lipid-nanoparticle-mediated intracellular mRNA delivery and function. Phys. Rev. E.

[250] Hatit M.Z.C., Dobrowolski C.N., Lokugamage M.P., Loughrey D., Ni H., Zurla C., Da Silva Sanchez A.J., Radmand A., Huayamares S.G., Zenhausern R. (2023). Nanoparticle stereochemistry-dependent endocytic processing improves in vivo mRNA delivery. Nat. Chem..

[251] Miao H., Huang K., Li Y., Li R., Zhou X., Shi J., Tong Z., Sun Z., Yu A. (2023). Optimization of formulation and atomization of lipid nanoparticles for the inhalation of mRNA. Int. J. Pharm..

[252] Hunter M.R., Cui L., Porebski B.T., Pereira S., Sonzini S., Odunze U., Iyer P., Engkvist O., Lloyd R.L., Peel S. (2023). Understanding Intracellular Biology to Improve mRNA Delivery by Lipid Nanoparticles. Small Methods.

[253] Yu X., Yu C., Wu X., Cui Y., Liu X., Jin Y., Li Y., Wang L. (2023). Validation of an HPLC-CAD Method for Determination of Lipid Content in LNP-Encapsulated COVID-19 mRNA Vaccines. Vaccines.

[254] Aliakbarinodehi N., Gallud A., Mapar M., Wesén E., Heydari S., Jing Y., Emilsson G., Liu K., Sabirsh A., Zhdanov V.P. (2022). Interaction Kinetics of Individual mRNA-Containing Lipid Nanoparticles with an Endosomal Membrane Mimic: Dependence on pH, Protein Corona Formation, and Lipoprotein Depletion. ACS Nano.

[255] Xue L., Gong N., Shepherd S.J., Xiong X., Liao X., Han X., Zhao G., Song C., Huang X., Zhang H. (2022). Rational Design of Bisphosphonate Lipid-like Materials for mRNA Delivery to the Bone Microenvironment. J. Am. Chem. Soc..

[256] Van Rijn C.J.M., Vlaming K.E., Bem R.A., Dekker R.J., Poortinga A., Breit T., van Leeuwen S., Ensink W.A., Van Wijnbergen K., Van Hamme J.L. (2023). Low energy nebulization preserves integrity of SARS-CoV-2 mRNA vaccines for respiratory delivery. Sci. Rep..

[257] Gan Z., Lokugamage M.P., Hatit M.Z.C., Hatit M.Z.C., Loughrey D., Paunovska K., Sato M., Cristian A., Dahlman J.E. (2020). Nanoparticles containing constrained phospholipids deliver mRNA to liver immune cells in vivo without targeting ligands. Bioeng. Transl. Med..

[258] Saunders K.O., Pardi N., Parks R., Santra S., Mu Z., Sutherland L., Scearce R., Barr M., Eaton A., Hernandez G. (2021). Lipid nanoparticle encapsulated nucleoside-modified mRNA vaccines elicit polyfunctional HIV-1 antibodies comparable to proteins in nonhuman primates. NPJ Vaccines.

[259] Shepherd S.J., Warzecha C.C., Yadavali S., El-Mayta R., Alameh M.G., Wang L., Weissman D., Wilson J.M., Issadore D., Mitchell M.J. (2021). Scalable mRNA and siRNA Lipid Nanoparticle Production Using a Parallelized Microfluidic Device. Nano Lett..

[260] Ye Z., Chen J., Zhao X., Li Y., Harmon J., Huang C., Chen J., Xu Q. (2022). In Vitro Engineering Chimeric Antigen Receptor Macrophages and T Cells by Lipid Nanoparticle-Mediated mRNA Delivery. ACS Biomater. Sci. Eng..

[261] Bepperling A., Richter G. (2023). Determination of mRNA copy number in degradable lipid nanoparticles via density contrast analytical ultracentrifugation. Eur. Biophys. J..

[262] Sarode A., Patel P., Vargas-Montoya N., Allawzi A., Zhilin-Roth A., Karmakar S., Boeglin L., Deng H., Karve S., DeRosa F. (2023). Inhalable dry powder product (DPP) of mRNA lipid nanoparticles (LNPs) for pulmonary delivery. Drug Deliv. Transl. Res..

[263] Huang H., Zhang C., Yang S., Xiao W., Zheng Q., Song X. (2021). The investigation of mRNA vaccines formulated in liposomes administrated in multiple routes against SARS-CoV-2. J. Control Release.

[264] Elia U., Ramishetti S., Rosenfeld R., Dammes N., Bar-Haim E., Naidu G.S., Makdasi E., Yahalom-Ronen Y., Tamir H., Paran N. (2021). Design of SARS-CoV-2 hFc-Conjugated Receptor-Binding Domain mRNA Vaccine Delivered via Lipid Nanoparticles. ACS Nano.

[265] Yang D., Song C.Q. (2023). The Delivery of ABE mRNA to the Adult Murine Liver by Lipid Nanoparticles (LNPs). Methods Mol. Biol..

[266] Nakashima I., Saito S., Akahoshi E., Yagyu S., Sugano-Ishihara M., Nakazawa Y. (2022). Non-viral inducible caspase 9 mRNA delivery using lipid nanoparticles against breast cancer: An in vitro study. Biochem. Biophys. Res. Commun..

[267] Wang T., Sung T.C., Yu T., Lin H.Y., Chen Y.H., Zhu Z.W., Gong J., Pan J., Higuchi A. (2023). Next-generation materials for RNA-lipid nanoparticles: Lyophilization and targeted transfection. J. Mater. Chem. B.

[268] Takanashi A., Pouton C.W., Al-Wassiti H. (2023). Delivery and Expression of mRNA in the Secondary Lymphoid Organs Drive Immune Responses to Lipid Nanoparticle-mRNA Vaccines after Intramuscular Injection. Mol. Pharm..

